# Investigating the Use of Telemedicine by Health Care Providers to Diagnose and Manage Patients With Musculoskeletal Disorders: Systematic Review and Meta-Analysis

**DOI:** 10.2196/52964

**Published:** 2024-09-23

**Authors:** Raphaël Vincent, Maxime Charron, Simon Lafrance, Audrey-Anne Cormier, Dahlia Kairy, François Desmeules

**Affiliations:** 1 School of Rehabilitation Faculty of Medicine Université de Montréal Montréal, QC Canada; 2 Hôpital Maisonneuve-Rosemont Research Center Université de Montréal Affiliated Research Center Montréal, QC Canada; 3 Centre for Interdisciplinary Research in Rehabilitation of Greater Montreal Institut Universitaire sur la Réadaptation en Déficience Physique de Montréal Montréal, QC Canada

**Keywords:** telemedicine, musculoskeletal diseases, physical examination, diagnosis, treatment, health care, telecare, meta-analysis, systematic review, telehealth, orthopedic, test, musculoskeletal disorder, MSKD, older adult, older adults, older person, older people, aging, musculoskeletal, mobile phone

## Abstract

**Background:**

Access to care is a major challenge for patients with musculoskeletal disorders (MSKDs). Telemedicine is one of the solutions to improve access to care. However, initial remote diagnosis of MSKDs involves some challenges, such as the impossibility of touching the patient during the physical examination, which makes it more complex to obtain a valid diagnosis. No meta-analysis has been performed to date to synthesize evidence regarding the initial assessment including a physical evaluation using telemedicine to diagnose patients with MSKDs.

**Objective:**

This study aims to appraise the evidence on diagnostic and treatment plan concordance between remote assessment using synchronous or asynchronous forms of telemedicine and usual in-person assessment for the initial evaluation of various MSKDs.

**Methods:**

An electronic search was conducted up to August 2023 using terms related to telemedicine and assessment of MSKDs. Methodological quality of studies was assessed with the Quality Assessment of Diagnostic Accuracy Studies 2 tool. Random-effect model meta-analyses were performed. The Grading of Recommendations, Assessment, Development, and Evaluations framework was used to synthesize the quality and certainty of the evidence.

**Results:**

A total of 23 concordance studies were eligible and included adult participants (N=1493) with various MSKDs. On the basis of high certainty, pooled κ and prevalence-adjusted and bias-adjusted κ for the diagnostic concordance between remote and in-person assessments of MSKDs were 0.80 (95% CI 0.72-0.89; 7 studies, 353 patients) and 0.83 (95% CI 0.76-0.89; 6 studies, 306 patients). On the basis of moderate certainty, pooled Gwet AC1 for treatment plan concordance between remote and in-person assessments of MSKDs was 0.90 (95% CI 0.80-0.99; 2 studies, 142 patients).

**Conclusions:**

The diagnostic concordance for MSKDs is good to very good. Treatment plan concordance is probably good to excellent. Studies evaluating the accuracy to detect red and yellow flags as well as the potential increase in associated health care resources use, such as imaging tests, are needed.

## Introduction

### Background

Almost 1.7 billion people have a musculoskeletal disorder (MSKD) in the world [1]. MSKDs refer to disorders affecting muscles, bones, and joints, such as low back pain, osteoarthritis, or tendinopathy [2]. MSKDs can lead to pain, disability, and poor health-related quality of life, resulting in a significant burden to health care systems as well as to society [2-6]. Evidence shows that a valid diagnosis through a careful initial evaluation and prompt treatment are essential for MSKDs [2,7]. Recommendations for various MSKDs emphasize the importance of screening for signs and symptoms of underlying serious pathologies (red flags) and for psychological factors associated with a poorer prognosis (yellow flags) during the initial evaluation as well as conducting a physical examination that includes measurements of mobility, strength, and use of orthopedic tests [2,8].

Significant health inequalities exist between urban and low-density population areas in several countries, with rural populations having a higher rate of injury and a higher risk of chronic MSKDs [9-11]. These inequalities can partially be explained by difficulties in accessing primary and secondary care [12-14]. The COVID-19 pandemic exacerbated these difficulties, with nearly 1 in 5 individuals unable to access health care during the first year of the COVID-19 pandemic, and patients with MSKDs were more affected by these delays than other patient populations [6,15-17]. Access to care is a major challenge for patients with MSKDs as delays can negatively impact clinical outcomes, such as pain, disability, or quality of life, while also potentially exacerbating psychological distress [18].

Telemedicine has been shown to have many benefits for patients, health care systems, and society [19]. It is an interesting option for optimizing health care access by removing unnecessary hurdles, such as geographical location or for patients with impaired mobility [19-21]. Telemedicine first appeared under asynchronous forms where communication between parties was not happening in real time [22]. Asynchronous forms are still used today for communication between patients and health care providers via messaging systems (emails and instant SMS text messaging) or smartphone apps for follow-up or counselling [23]. Synchronous forms of telemedicine with phone calls and videoconferencing allow real-time interaction between parties [22]. The use of videoconferencing in telemedicine has been a major progress by allowing visual evaluation of movement, edema, or scars, for example, or to observe patient physical performance and function [24]. The use of telemedicine in high-income countries now mainly focuses on patient remote management or follow-ups of patients who have already been assessed in person [25]. Telemedicine is well implanted in medical specialties, such as radiology, dermatology, or psychiatry, but is not widely used in the MSKDs care pathways, particularly for the initial assessment of new patients where a diagnosis is required [25,26]. The use of telemedicine for MSKDs brings many challenges related to the geographical distancing of the patient and the health care provider [27-31]. The loss of physical contact during the physical examination and treatments raises questions among patients and clinicians about the relevance, safety, and effectiveness of remote care [27,30,31]. Patients and health care providers also express concerns about the quality of the therapeutic relationship when consultations are not conducted in person [27,28]. However, telemedicine offers advantages to patients and health care providers, such as better accessibility, greater flexibility, and the possibility to offer interventions that are adapted to a patient’s environment as the remote consultation will likely be in a person’s home [27].

Previous systematic reviews and meta-analyses have reported that remote patient follow-up management after an in-person initial evaluation is a valid alternative to usual in-person management and leads to similar benefits in clinical outcomes for various MSKDs, such as low back pain, neck pain, shoulder pain, or neurological conditions [32-35]. Previous systematic reviews focusing on synchronous remote initial assessment highlighted that most clinical measures remotely evaluated have a good concurrent validity and an excellent reliability [24,36]. Remote diagnoses were highly concordant with in-person diagnoses, but no meta-analysis was performed on these results, and these systematic reviews only included studies with physiotherapists as evaluators, excluding other health care providers who usually assess patients with MSKDs, such as orthopedic surgeons or primary care physicians [36]. As the diagnosis as well as the treatment plan are key elements of an initial assessment, it is crucial to undertake a comprehensive appraisal, including a meta-analysis of the concordance between remote and in-person assessment for new patients with MSKDs.

### Objective

This systematic review with meta-analysis aimed to appraise the available evidence on diagnosis and care concordance after an initial assessment between a remote evaluation and an in-person evaluation for the evaluation of various MSKDs.

Therefore, the research question for this systematic review was as follows: Can a complete, valid, and safe assessment be carried out using asynchronous, synchronous or both forms of telehealth to diagnose various MSKDs?

## Methods

### Protocol, Registration, and Deviation

The protocol of this systematic review has been registered on PROSPERO (CRD42022335606) [37]. A total of 2 deviations from the protocol have occurred and are as follows: (1) We now only present results for the first specific objective (concordance of diagnosis and management) in this publication, as there were too much data concerning the second objective (concordance of clinical measures) to be presented in 1 publication (data concerning the second objective will be presented in a second publication); (2) We have now added the calculation of prediction intervals to further assess the heterogeneity of the pooled estimates in addition to the *I*^2^ and τ² statistics.

### Literature Search

An electronic search was conducted in 4 databases (MEDLINE, Embase, Cochrane Central, and CINAHL) from January 2000 to August 2023 using terms related to telemedicine by any type of health care providers (physicians, orthopedic surgeons, physiotherapists, or other providers), MSKDs, and the assessment or the clinical examination of these conditions. A trained librarian was consulted during the development of our research strategy. We chose to limit our search to the last 2 decades to identify technologies still in use and readily available to clinicians. The full search strategy is available in the Multimedia Appendix 1. Reference lists of identified published studies and previous systematic reviews were checked for any additional studies.

### Study Selection

A pair of reviewers (RV and MC or AAC) independently reviewed titles and abstracts to identify studies of interest. Consensus from reviewers was required to include studies. A third reviewer (SL) was available if a consensus was not achieved by the 2 initial reviewers. Textbox 1 shows the inclusion criteria for articles.

**Inclusion criteria for eligible studies.**

**Inclusion criteria**

- Participants with symptoms related to suspected musculoskeletal disorders.
- Participants assessed during a remote evaluation (synchronous or asynchronous) and compared with an in-person evaluation by any health care provider.
- Any type of diagnostic, care or treatment plan concordance outcomes (such as raw agreement, κ, prevalence-adjusted and bias-adjusted κ, or other concordance coefficients).
- Technologies used for the remote assessment had to be accessible for routine clinical use (no experimental setup and no use of sensors to be placed on the participants).
- Full-article or conference abstracts written in French or English.

### Data Extraction

Data of included studies were extracted using a predefined standardized form documenting the following: authors’ names, year of publication, study design, country, care settings, characteristics of the remote assessment (modality and technology used as well as the presence of a third party to help with patient evaluation), body region or regions affected, number of participants, participant characteristics, health care provider characteristics, and outcome measures. Data extraction was performed by 1 evaluator (RV), and the extracted data were reviewed and verified by a second evaluator (MC or AAC). When data were missing or incomplete, attempts to contact original authors were made to obtain complete data and results.

### Methodological Quality Assessment

Methodological quality of included studies was assessed with the valid and reliable Quality Assessment of Diagnostic Accuracy Studies 2 (QUADAS-2) tool [38]. This tool appraises patient selection, index tests, reference standard, flow, and timing. QUADAS-2 also assesses the presence of applicability concerns that would decrease the external validity of the study results. Assessment of methodological quality and applicability concerns was performed by a pair of independent evaluators (RV and MC or SL or AAC or Claudia Cosculluela); the final score was obtained through consensus. In case of disagreement, a third evaluator was available to facilitate consensus (MC or SL). Studies were considered at risk of bias if at least 1 item of the QUADAS-2 was evaluated at high risk of bias.

### Data Synthesis

κ, prevalence-adjusted and bias-adjusted κ (PABAK), and Gwet AC1 coefficients were pooled into separate meta-analyses. Weighted means were calculated for raw agreement. Random-effect model meta-analyses were performed using the *metafor* package in RStudio Team (2020, Rstudio: Integrated Development for R. Rstudio) [39,40]. Secondary analyses for different modalities of delivery (asynchronous or synchronous) and for affected body regions were performed. Sensitivity analyses were also performed, including only studies with a low risk of bias (with no item ranked as high risk of bias on the QUADAS-2). Pooled concordance estimates were interpreted as follows: 0.00-0.20=weak, 0.21-0.40=slight, 0.41-0.60=moderate, 0.61-0.80=good, 0.81-0.90=very good, and 0.91-1.00=excellent agreement [41,42]. Coding scripts are available in Multimedia Appendix 2. For all meta-analyses, α levels were set at .05, and 95% CIs were calculated. Statistical heterogeneity was quantified and reported using the *I*^2^ and τ² statistics and interpreted according to the Cochrane methodology [43]. Prediction intervals were also calculated using the Comprehensive Meta-Analysis software (version 4.0; H.Biostat) to further assess the heterogeneity of the pooled estimates [44,45]. A narrative synthesis was performed for studies and outcomes not pooled in meta-analyses or for conference abstracts included in this review.

The Grading of Recommendations, Assessment, Development, and Evaluations (GRADE) framework was used for grading the quality and certainty of evidence and for formulating recommendations [46]. Level of evidence was interpreted as follows: very low certainty: the true effect is probably markedly different from the pooled estimated effect, low certainty: the true effect might be markedly different from the pooled estimated effect, moderate certainty: we believe that the true effect is probably close to the pooled estimated effect, and high certainty: we are very confident that the true effect is similar to the pooled estimated effect.

## Results

### Overview

**Figure 1:**
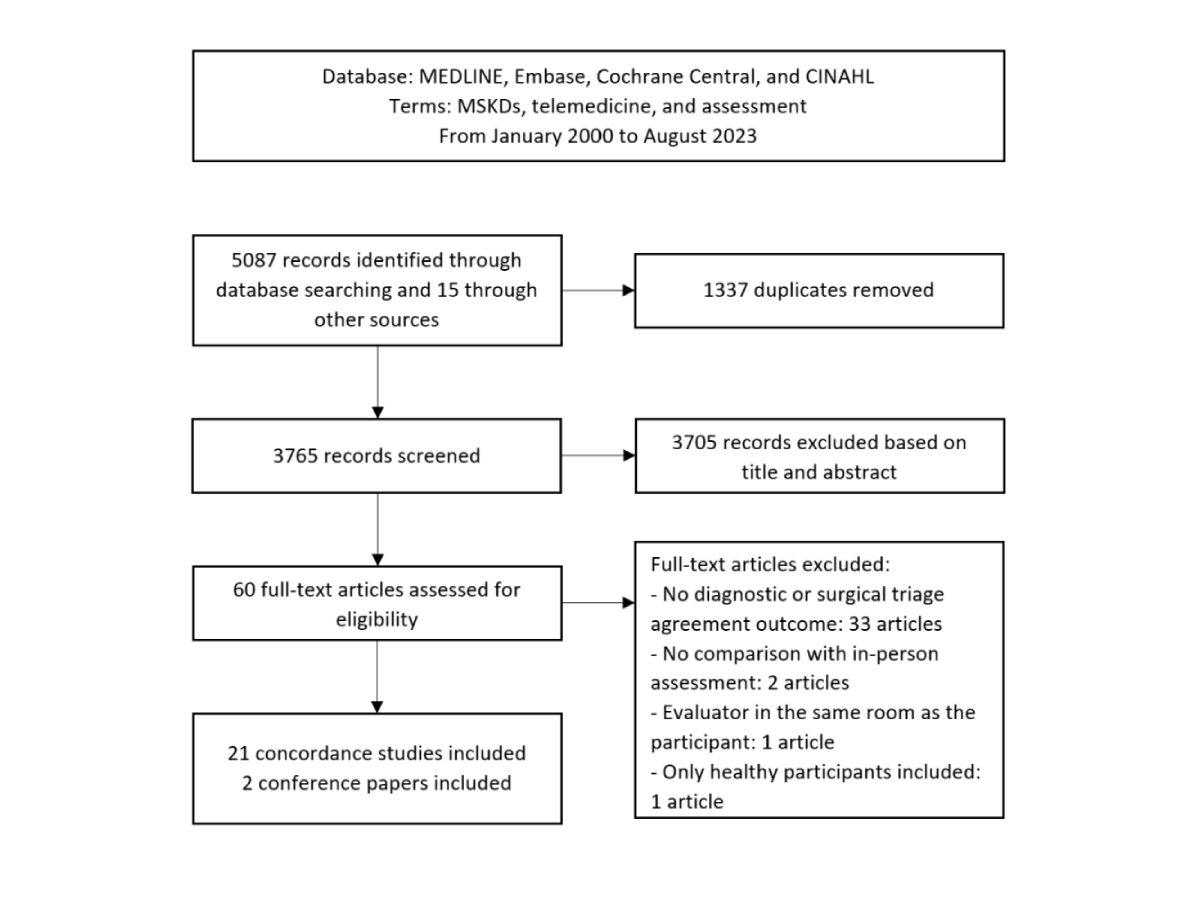
PRISMA (Preferred Reporting Items for Systematic Reviews and Meta-Analyses) flowchart for concordance studies selection.

After full-text review, 11 studies on in-person remote diagnostic concordance [42,47-56], 7 studies on in-person remote diagnostic and treatment plan concordance [41,57-62], and 3 studies on in-person remote treatment plan concordance [63-65] were included (Figure 1). No studies presented results on triage concordance of surgical candidates. A total of 2 conference abstracts were also included on in-person remote diagnostic concordance [66,67]. Reasons for exclusions are available in Multimedia Appendix 3. Full characteristics of the included studies are presented in Tables 1 and 2 and Table S1 in Multimedia Appendix 4.

**Table 1:** Characteristics of the included studies (study settings).

Author and year	Design	Country	Care setting	Modality of assessment	Technology
Abboud et al [57], 2005	Prospective cohort	United States	Orthopedic	Asynchronous	Recorded photographs and data
Bovonratwet et al [58], 2022	Retrospective cohort	United States	Orthopedic	Synchronous	Videoconferencing
Bradley et al [47], 2021	Case control	United States	Orthopedic	Synchronous	Videoconferencing
Demaerschalk et al [48], 2022	Retrospective cohort	United States	Clinic	Synchronous	Videoconferencing
Dias et al [56], 2023	Prospective cohort	Brazil	Orthopedic	Synchronous	Videoconferencing
Exposto et al [42], 2022	Prospective cohort	Denmark	Dentistry	Synchronous	Videoconferencing
Melnick et al [65], 2023	Retrospective cohort	United States	Orthopedic	Synchronous	Videoconferencing
Michaelson et al [62], 2023	Retrospective cohort	United States	Orthopedic	Synchronous	Videoconferencing with the presence of the patient’s caregiver
Rabin et al [61], 2022	Prospective cohort	Israel	Orthopedic	Synchronous	Videoconferencing with presence of a research assistant or the patient’s caregiver
Tachakra et al [54], 2000	Prospective cohort	United Kingdom	Emergency Department	Synchronous	Videoconferencing with presence of a nurse
Wang et al [55], 2022	Prospective cohort	United States	Orthopedic	Asynchronous	Recorded video of a standardized clinical examination with presence of a research assistant
Cottrell et al [59], 2018	Prospective cohort	Australia	Physiotherapy	Synchronous	Videoconferencing
Lade et al [49], 2012	Prospective cohort	Australia	Physiotherapy	Synchronous	Videoconferencing
Lovo et al [41], 2022	Prospective cohort	Canada	Rural clinic	Synchronous	Videoconferencing with presence of a nurse
Peterson et al [63], 2019	Prospective cohort	United States	Physiotherapy	Synchronous	Videoconferencing
Richardson et al [50], 2017	Prospective cohort	Australia	Physiotherapy	Synchronous	Videoconferencing
Russell et al [52], 2010	Prospective cohort	Australia	Physiotherapy	Synchronous	Videoconferencing
Russell et al [51], 2010	Prospective cohort	Australia	Physiotherapy	Synchronous	Videoconferencing
Steele et al [53], 2012	Prospective cohort	Australia	Physiotherapy	Synchronous	Videoconferencing
Turner et al [60], 2019	Prospective cohort	United Kingdom	Physiotherapy	Synchronous	Telephone
Worboys et al [64], 2018	Prospective cohort	Australia	Occupational therapy	Synchronous	Videoconferencing with presence of an assistant

**Table 2:** Characteristics of the included studies (patient and evaluator characteristics).

Author and year	Patients					Evaluators			Access to imaging tests to make diagnosis
	Musculoskeletal disorders (MSKDs^a^)	Values, n	Age (y), mean (SD)	Female (%)	Profession (number of evaluators)		Training		
Abboud et al [57], 2005	Upper extremity MSKDs	100	43 (NR^b^)	50	Orthopedic surgeons (n=3)		NR	Yes	
Bovonratwet et al [58], 2022	Spine MSKDs	65	57.5 (14.8)	46	Orthopedic surgeons (n=5)		NR	Yes	
Bradley et al [47], 2021	Shoulder MSKDs	50	58.2	53	Orthopedic surgeons (n=9)		NR	No	
Demaerschalk et al [48], 2022	MSKDs	300	NR	NR	Physicians, nurses, and physician assistants		NR	NR	
Dias et al [56], 2023	Low back pain	122	47.8 (13.1)	66	Orthopedic surgeons (n=17)		NR	NR	
Exposto et al [42], 2022	Temporomandibular joint disorders	15	31 (12)	75	Dentists (n=5)		10 hours of training	NR	
Melnick et al [65], 2023	Spine MSKDs	152	61.4 (15.4)	43	Orthopedic surgeons (n=7)		NR	NR	
Michaelson et al [62], 2023	Shoulder MSKDs	82	52.6 (NR)	NR	Orthopedic surgeons (n=5)		NR	Yes	
Rabin et al [61], 2022	Shoulder MSKDs	47	44.6 (22)	36	Orthopedic surgeons (n=2)		4 hours of meeting and training with 5 pilot test participants	Yes	
Tachakra et al [54], 2000	Minor trauma injuries	200	NR	31	Emergency physician		NR	Yes	
Wang et al [55], 2022	Shoulder MSKDs	32	50.2 (16.2)	47	Orthopedic surgeon (n=1)		NR	Yes	
Cottrell et al [59], 2018	Chronic MSKDs	42	52.7 (14.5)	57	Advanced practice physiotherapists (n=6)		Training with 4 pilot test participants	Yes	
Lade et al [49], 2012	Elbow MSKDs	10	38 (13)	10	Physiotherapy students (n=3)		Support by an experienced physiotherapist	NR	
Lovo et al [41], 2022	Chronic back pain	27	53.7 (18.1)	70	Physiotherapists (n=2) and nurses (n=2)		Interprofessional training before the study	NR	
Peterson et al [63], 2019	Low back pain	47	48.6 (15)	70	Physiotherapists (n=2)		NR	NR	
Richardson et al [50], 2017	Knee MSKDs	18	23 (7)	55	Physiotherapists (n=3)		Training with 2 pilot test participants	NR	
Russell et al [52], 2010	Ankle MSKDs	15	24.5 (10.8)	67	Physiotherapy students (n=3)		Training with 2 pilot test participants	NR	
Russell et al [51], 2010	Lower limb MSKDs (nonarticular)	19	26 (13)	74	Physiotherapists (n=3)		Training with 2 pilot test participants	NR	
Steele et al [53], 2012	Shoulder MSKDs	22	30.7 (14.2)	27	Physiotherapy students (n=3)		Support by an experienced physiotherapist	NR	
Turner et al [60], 2019	MSKDs	55	NR	NR	Physiotherapists (n=22)		NR	NR	
Worboys et al [64], 2018	Hand injuries	18	NR	45	Occupational therapists (n=4)		NR	NR	

^a^MSKD: musculoskeletal disorder.

^b^NR: not reported.

### Countries and Clinical Settings

Studies were conducted in Australia (7/23, 30%), the United States (8/23, 35%), the United Kingdom (4/23, 17%), Denmark (1/23, 4%), Canada (1/23, 4%), Brazil (1/23, 4%), and Israel (1/23, 4%). In terms of care settings, studies were conducted in physiotherapy primary care clinics (8/23, 35%) [49-53,59,60,63], in orthopedic specialized care (8/23, 35%) [47,55-58,61,62,65], and in primary medical care (4/23, 17%) [41,48,66,67]; one study was conducted in an emergency department [54]. One study was concerning temporomandibular disorders in dentistry [42], and 1 study was conducted in an occupational therapy clinic [64]. Synchronous modalities were the most frequent way of contacting patients (21/23, 91%), with 18 (78%) of the 23 studies using a videoconferencing interface [41,42,47-56,58,59,61-63,65] and 3 (13%) using telephone calls [60,66,67]. Support staff was present to help with patient assessment and mobilization during the remote evaluation in 5 (22%) of the 23 studies [41,54,55,61,65]. Asynchronous modalities were used in 2 (8%) of the 23 studies, 1 using photographs of the patients [57] and 1 using video recordings of a self-performed clinical examination under the supervision of an assistant [55].

### Participants

A total of 1849 participants were included. Mean age of participants was 49.6 (SD 14.9) years. Female gender accounted for 48% (887/1849) of the participants. Included participants had spinal disorders (703/1849, 38.02%), upper limb disorders (375/1849, 20.3%), lower limb disorders (201/1849, 10.9%), temporomandibular disorders (15/1849, 0.8%), and unspecified various MSKDs (555/1849, 30%). Of the 23 studies, 5 (22%) were conducted in university physiotherapy and dentistry clinics, which included a younger sample of patients and more acute or traumatic pathologies [42,50-53]. Other studies were conducted essentially in usual orthopedic and physiotherapy clinics and included broader samples with various MSKDs clinical representations.

### Evaluators and Training

Evaluators were physiotherapists in 35% (8/23) of the studies, physiotherapy students in 13% (3/23) of the studies, or orthopedic surgeons in 35% (8/23) of the studies. Other health care providers involved were nurses, dentists, occupational therapists, and emergency and primary care physicians. In 7 (30%) of the 23 studies, evaluators received specific training on how to perform and adapt their remote assessment [41,42,50-52,59,61]. No information regarding specific training was available for the remaining studies [47,48,54-58,60,62-67]. Of the 23 studies, 3 (13%) involving physiotherapy students allowed them to be advised by a senior physiotherapist if they felt the need [49,52,53]. In 7 (30%) of the 23 studies, evaluators had access to participants’ imaging test results [54,55,57-59,61,62] while in 3 (13%) studies they did not [47,66,67]. No information regarding access to imaging results was available for the remaining studies.

### Methodological Quality of Included Studies

Of the 21 studies, 3 (14%) had a perfect QUADAS-2 score [55-57]. A total Of the 21 studies, 11 (52%) had at least 1 item of the QUADAS-2 scored as a high a risk of bias [42,47,49,52-54,58,59,62,63,65] and 9 (43%) had at least 1 item of the QUADAS-2 score scored with an unclear risk of bias [41,48,49,51-54,58,61]. The main shortcomings concerned recruitment method or patient selection [50-52,58,59,61,63,64], the use of case-control study design [42,47], and the lack of blinding or validity of the evaluators’ diagnosis (diagnoses made by physiotherapy students with inconsistent supervision) [49,52-54,62,65]. Of the 9 (43%) had an applicability concern related to the representativeness of the sample of patients recruited [50-53,58,60,61,63,64]. A study had applicability concerns due to the choice of the index test and of the reference standard for the remote and in-person assessments because the remote and in-person assessments were conducted simultaneously [64]. Details are presented in Table 3.

**Table 3:** Quality of the included concordance studies based on the Quality Assessment of Diagnostic Accuracy Studies 2 Tool.

Author and year	Risk of bias					Applicability concerns		
	Patient selection	Index test	Reference standard	Flow and timing	Patient selection		Index test	Reference standard
Abboud et al [57], 2005	Low	Low	Low	Low	Low		Low	Low
Dias et al [56], 2023	Low	Low	Low	Low	Low		Low	Low
Wang et al [55], 2022	Low	Low	Low	Low	Low		Low	Low
Bovonratwet et al [58], 2022	High^a^	Low	Unclear	Low	High^a^		Low	Low
Bradley et al [47], 2021	High^b^	Low	Low	Low	Low		Low	Low
Cottrell et al [59], 2018	High^c^	Low	Low	Low	Low		Low	Low
Demaerschalk et al [48], 2022	Low	Low	Unclear	Low	Low		Low	Low
Exposto et al [42], 2022	High^b^	Low	Low	Low	Low		Low	Low
Lade et al [49], 2012	Unclear	Low	High^d^	Low	Low		Low	Low
Lovo et al [41], 2022	Unclear	Low	Low	Low	Low		Low	Low
Melnick et al [65], 2023	Low	Low	High^e^	Low	Low		Low	Low
Michaelson et al [62], 2023	Low	Low	High^e^	Unclear	Low		Unclear	Low
Peterson et al [63], 2019	High^f,g^	Low	Low	Low	High^g^		Low	Low
Rabin et al [61], 2022	Low	Unclear	Unclear	Low	High^h^		Low	Low
Richardson et al [50], 2017	Low	Low	Low	Low	High^i^		Low	Low
Russell et al [52], 2010	Unclear	Low	High^d^	Low	High^i^		Low	Low
Russell et al [51], 2010	Unclear	Low	Low	Low	High^i^		Low	Low
Steele et al [53], 2012	Unclear	Low	High^d^	Low	High^i^		Low	Low
Tachakra et al [54], 2022	Unclear	Low	High^j^	Low	Low		Low	Low
Turner et al [60], 2019	Low	Low	Low	Low	Unclear		Low	Low
Worboys et al [64], 2018	Low	Low	Low	Low	High^k^		High^k^	High^k^

^a^Patients who did not have a specific diagnosis and treatment plan during the remote assessment were excluded, which could increase agreement by including only patients who could be easily assessed remotely.

^b^Case-control design was used in the study.

^c^Only a convenience sampling was used for recruitment.

^d^Assessments were conducted by physiotherapy students, which could reduce the validity of the diagnoses made by evaluators.

^e^During the in-person assessment, evaluators had access to the treatment plan proposed after the remote assessment.

^f^Only a convenience sampling was used for recruitment.

^g^Patients with surgical history of the lumbosacral spine, visible deformity, or no reproduction of symptoms with certain orthopedic tests were excluded.

^h^Almost half of the patients (22/47, 47%) were evaluated during a postoperative or nonoperative follow-up consultation, which could increase agreement on diagnosis and treatment plan.

^i^This cohort was composed of younger patients recruited in the university clinic with only acute or subacute injuries, without degenerative pathologies.

^j^The same evaluator conducted the remote and in-person assessments.

^k^Initial assessments were excluded, and a third party was present with the patient to assist with data collection, which could have increased the agreement.

### Diagnostic Concordance Between Remote and In-Person Assessments

**Figure 2:**
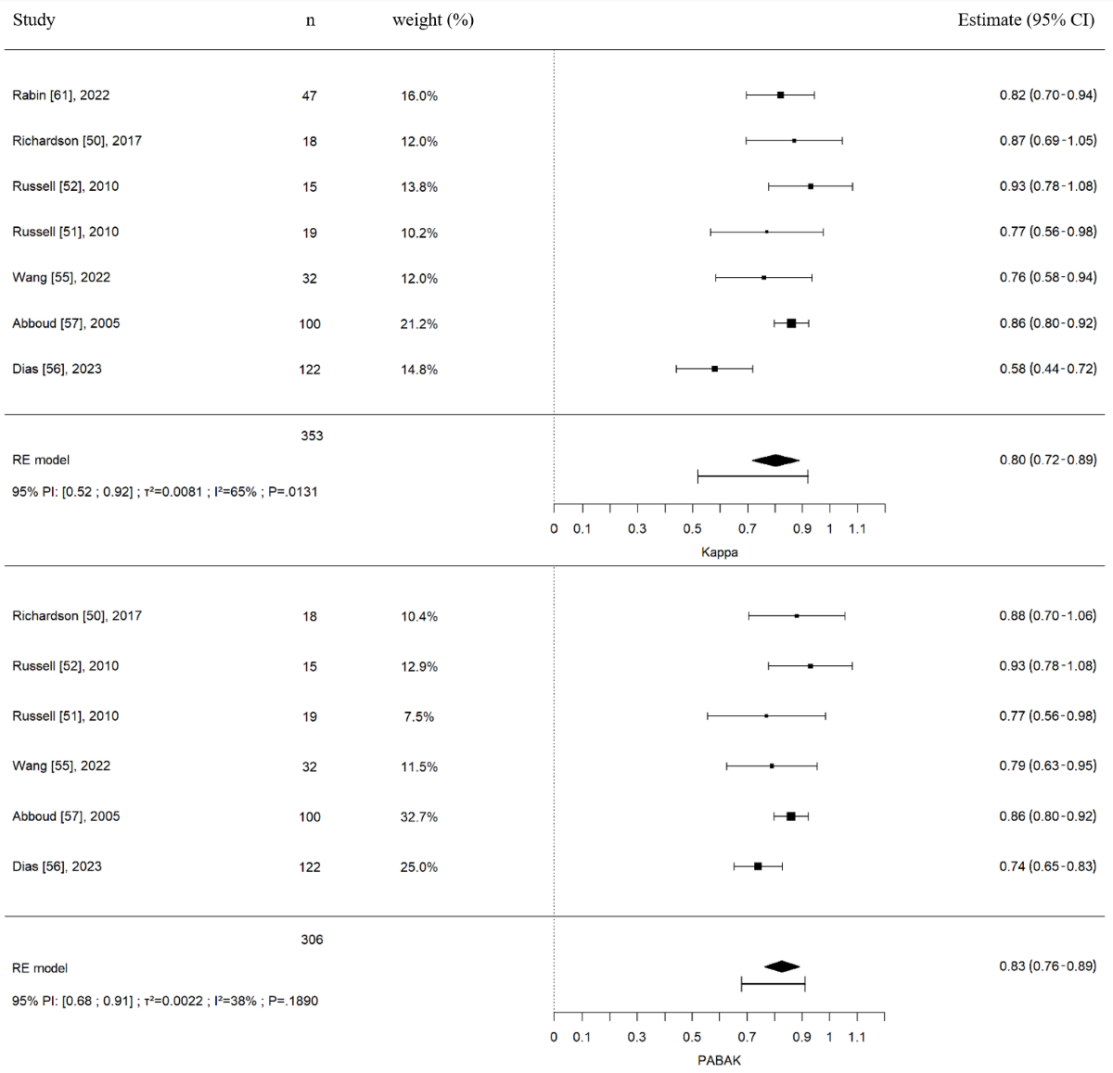
Diagnostic concordance (Cohen κs and prevalence-adjusted and bias-adjusted κs [PABAK]) between in-person and remote assessment in patients with musculoskeletal disorders. Meta-analysis with pooled Cohen κs included studies with physiotherapists (6/32, 19%), orthopedic surgeons (23/32, 72%), and physiotherapy students (3/32, 9%) as evaluators. Meta-analysis with pooled PABAKs included studies with physiotherapists (6/30, 33%), orthopedic surgeons (21/30, 50%) and physiotherapy students (3/30, 17%) as evaluators. 95% PI: 95% prediction interval; RE: random-effect model; τ^2^/I^2^/P: test for heterogeneity [50-52,55-57,61].

Of the 23 studies, 17 (74%) reported diagnostic concordance results between in-person and remote assessments, and a study [62] reported diagnostic changes between in-person and remote assessments; the overall raw agreement was 85.9% (1352/1574 patients). Of the 23 studies, diagnostic concordance between in-person and remote assessments pooled estimations were possible for 7 (30%) studies for κ and 6 (26%) for PABAK estimates. The pooled estimates are presented in Figure 2. The pooled κ was 0.80 (95% CI 0.72-0.89; 7 studies, 353 patients), and the pooled PABAK was 0.83 (95% CI 0.76-0.89; 6 studies, 306 patients), corresponding to very good agreement between both types of assessments. Statistical heterogeneity measured with the *I*^2^ statistic were 65% and 38%, respectively, and considered potentially moderate for these meta-analyses. Prediction intervals for the pooled κ and PABAK were (0.52-0.92) and (0.68-0.91), which were very similar to the calculated CIs for the PABAK but not for the κ, and that difference could be attributed to the κ estimate in the study by Dias et al [56].

Another study could not be pooled in previous analyses as Fleiss κs were used to evaluate the agreement between in-person and remote assessments [42]. Diagnostic concordance between in-person and remote assessments for the study by Exposto et al [42] for patients with temporomandibular disorders reported Fleiss κs between 0.52 and 1.0, depending on the specific temporomandibular disorders (myalgia of the masseter or the temporalis muscles, temporomandibular arthralgia, or disk displacement with reduction).

Subgroup analyses were performed according to the presence or not of a third party to evaluate the participant during the remote evaluation (Figure S1 in Multimedia Appendix 4), profession of the health care providers (Figures S2 and S3 in Multimedia Appendix 4), body regions (Figures S2 and S4 in Multimedia Appendix 4), and asynchronous or synchronous evaluation forms of telemedicine used (Figures S5 and S6 in Multimedia Appendix 4). Similar results for diagnostic concordance were reported for all these subgroup analyses. For body regions, subgroup analyses were performed specific to upper and lower limb MSKDs. Analyses excluding high risk-of-bias studies (studies with a QUADAS-2 without items evaluated at high risk of bias) reported similar results for diagnostic concordance (Figure S7 in Multimedia Appendix 4).

### Treatment Plan Concordance Between Remote and In-Person Assessments

**Figure 3:**
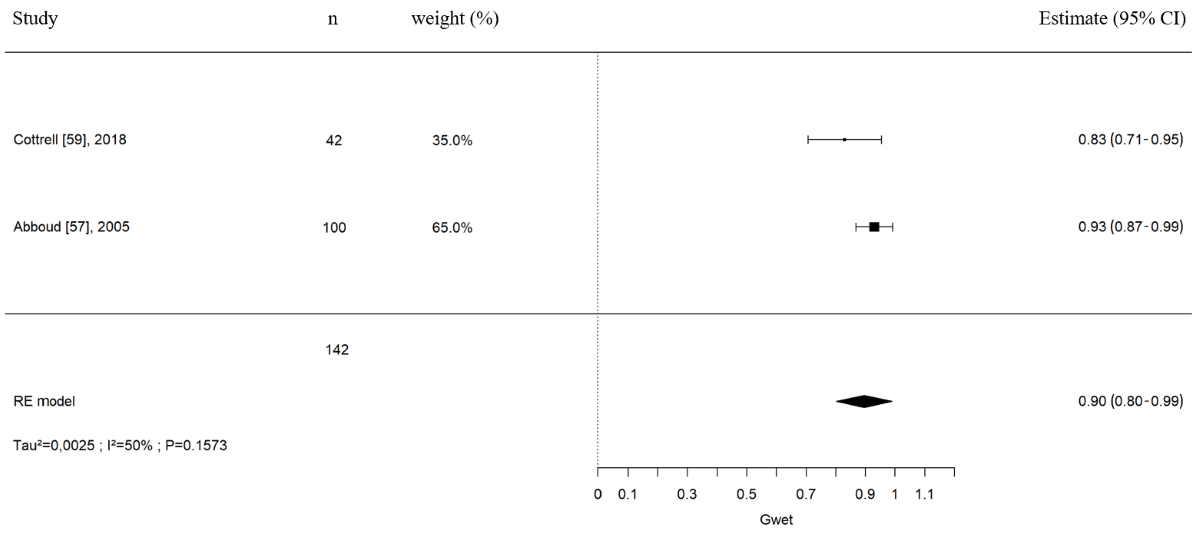
Treatment plan concordance (Gwet AC1 coefficients) between in-person and remote assessment in patients with musculoskeletal disorders. Meta-analysis with pooled Gwet AC1 coefficients included studies with advanced practice physiotherapists (6/9, 67%) and orthopedic surgeons (3/9, 33%). Gwet: Gwet AC1 coefficient; RE: random-effect model; τ^2^/I^2^/P: test for heterogeneity [57,59].

Of the 23 studies, 8 (35%) reported treatment plan concordance outcomes between in-person and remote assessments and 2 (9%) [62,65] reported treatment plan changes between in-person and remote assessments. Overall raw agreement was 84% (406/483 patients). Treatment plan concordance between in-person and remote assessments pooled estimations were only possible for 2 (9%) of the 23 studies for Gwet AC1 coefficient estimates. The pooled estimates are presented in Figure 3. The pooled Gwet AC1 coefficient was 0.90 (95% CI 0.80-0.99; 2 studies, 142 patients), corresponding to a good to excellent agreement between both types of assessments. Statistical heterogeneity (*I*^2^=50%) was potentially moderate for this meta-analysis. Prediction intervals could not be calculated as this meta-analysis is based on only 2 studies.

Of the 23 studies, 2 (9%) reporting κ estimates (2 studies, n=147) were not pooled together because of statistical heterogeneity (*I*^2^=94.79%). The first study by Abboud et al [57] compared the treatment plan concordance among 3 orthopedic surgeons for patients with upper limb MSKDs and reported an excellent agreement with a κ of 0.91 (95% CI 0.85-0.97; 100 patients). The other study by Rabin et al [61] compared the treatment plan concordance between 2 orthopedic surgeons for patients with shoulder pathologies. The authors reported only moderate agreement between remote and in-person proposed treatment plans with a κ of 0.43 (95% CI 0.22-0.64; 47 patients).

Another study was not pooled in these analyses as the evaluators were physiotherapists and had to choose among 3 physiotherapy treatments for low back pain patients (manipulation, specific exercises, or stabilization exercises). In the study by Peterson et al [63], the treatment plan concordance between in-person and remote assessments evaluated was only moderate with a κ of 0.52 (95% CI 0.32-0.72) and a PABAK of 0.52 (95% CI 0.31-0.72).

### GRADE Analysis

**Figure 4:**
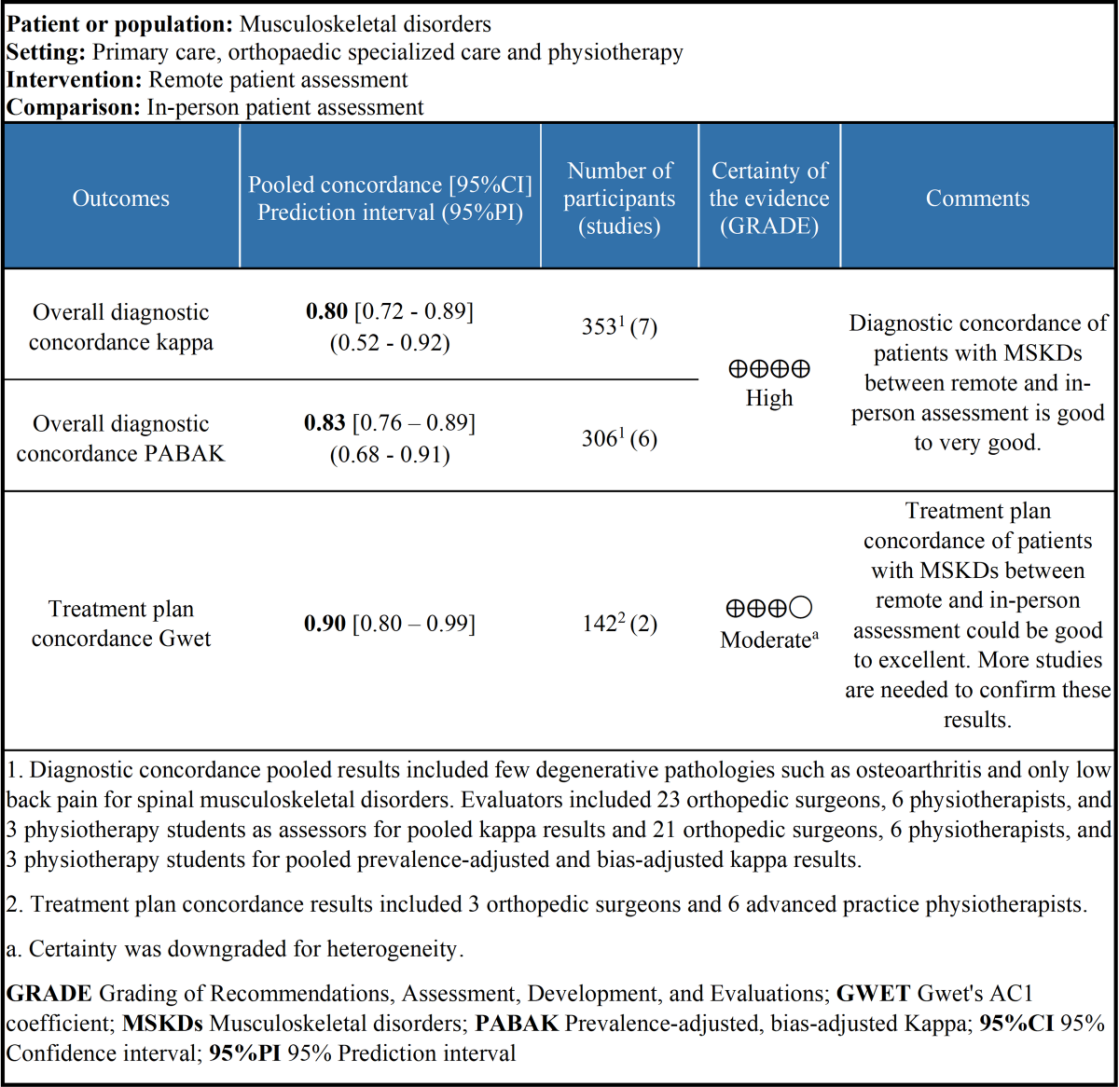
Summary of the findings and Grading of Recommendations, Assessment, Development, and Evaluations analysis for diagnostic and treatment plan concordance.

Pooled results for diagnostic concordance were of high certainty, highlighting a good to very good concordance between remote and in-person assessments (Figure 4).

Pooled results for treatment plan concordance were of moderate certainty, highlighting a good to excellent concordance between remote and in-person assessments (Figure 4). The *I*^2^ was at 50%, and prediction intervals could not be calculated; therefore, certainty of the results was downgraded.

## Discussion

### Principal Findings

The aim of this systematic review and meta-analysis was to appraise the available evidence on diagnosis and care concordance after an initial assessment between a remote evaluation and an in-person evaluation for the initial evaluation of various MSKDs. On the basis of our results, health care providers can remotely make a diagnosis that is concordant with a usual in-person assessment. These results are robust and among various populations with acute or chronic MSKDs; we conducted several secondary analyses to assess the impact of various factors that may affect the validity and the feasibility of a remote assessment. Presence of assistance with the patient, body region being assessed, technologies used, and different health care providers’ profession did not appear to influence the accuracy of the remote assessment, but these findings need to be confirmed due to the limited number of studies in certain analyses. Sensitivity analyses, including only studies without high risk of bias and results from the study by Exposto et al [42], not pooled in the meta-analysis, yielded similar results. The study by Dias et al [56] reported a lower κ than the rest of the pooled studies but a similar raw agreement and PABAK (not calculated in the original paper but extracted and calculated by our team). The distribution of diagnoses in the study by Dias et al [56] shows a very high prevalence of some diagnostic categories and a very low prevalence of others. This imbalance may have biased and lowered the κ statistical value [68,69]. Overall concordance was found to be high in our review, but it may still vary across certain MSKDs, and our secondary analyses with fewer study and participants may not have been able to capture this issue. Moreover, studies on various spinal disorders, such as neck pain, or on degenerative pathologies, such as osteoarthritis, which were only marginally represented in the included studies, but very prevalent, are also needed.

Treatment plans proposed remotely had a good to excellent concordance with the treatment plans proposed in person. It may seem logical that if the remote diagnosis is correct, the treatment plan would be similar. However, there is uncertainty about these results because overall less data were available on the treatment plan in the literature; only 2 studies could be pooled and showed potentially moderate heterogeneity. Raw agreement between remote and in-person treatment plan was 84% (406/483 patients; 9 studies), which can be considered high. Again, these results are promising but need to be confirmed by more studies using statistical estimates to conclude on treatment plan concordance.

However, it should be kept in mind that an initial assessment is not only a diagnosis and a treatment plan. Some other key elements were not or could not have been properly evaluated with a remote assessment in several of the included studies. No data are available on the detection of serious pathologies (red flags) or the evaluation psychosocial risk factors (yellow flags) using a remote assessment [24]. Most red or yellow flags are identified during the subjective examination by elements of the patient’s history, specific questions, or questionnaires [8,70]. They may also be identifiable during a remote assessment by using the same screening procedure as during an in-person assessment. However, some tests used to rule out the presence of serious pathologies cannot be performed remotely, such as certain tests of the neurological examination, and patients may be less likely to talk about the psychosocial problems they are experiencing during a remote video or phone encounter. Among the studies we included, none reported having identified serious pathologies. However, given the small sample sizes of the studies and the low prevalence of serious conditions, it is unlikely that several patients with a serious condition were in the included samples [8]. Caution is therefore warranted when concluding that a remote assessment is as safe as traditional in-person assessment and that patients with important psychosocial risk factors are accurately identified. This is the first systematic review with meta-analyses addressing the agreement between remote and in-person diagnoses and treatment plan for MSKDs. Our results are consistent with previous reviews investigating the validity and reliability of different parts of a remote physical examination for MSKDs. Previous reviews have highlighted that most clinical measures assessed by videoconferencing applications, such as pain (visual analog scale) or range of motion assessment in patients with MSKDs, have good concurrent validity and excellent reliability [24,36]. Nevertheless, some measures, such as orthopedic tests, which are usually hands on, have lower concurrent validity and reliability when assessed remotely or simply cannot be performed [47]. Adaptations to the usual in-person physical examination in the musculoskeletal field still need to be developed to achieve validity and reliability comparable to in-person physical examination or to establish that the accuracy of a remote assessment is not as high [24,36,71]. Our results are complementary to other reviews with meta-analyses conducted on remote management of patients with MSKDs that show that remote care after an in-person assessment leads to similar improvements in clinical outcomes as the usual fully in-person approach [32-35]. Guidelines for MSKDs management now emphasize the importance of patient education and self-management as well as physical activity and exercise prescription [2]; all these interventions can be performed remotely [72,73]. In conjunction with our conclusions, the actual body of evidence on remote patient follow-up and clinical measures as well as recommended interventions for MSKDs potentially support the benefits of full remote care pathways for patients with MSKDs.

As stated earlier, more evidence is still needed on the impact of remote assessment for MSKDs. Future studies should also focus on the potential impacts of an initial remote evaluation on health care resources use, such as the use of imaging and other paraclinical investigations, as telemedicine could increase their use. Clinicians and patients express doubts on the validity and safety of a complete remote evaluation because of the impossibility of a hands-on assessment [29,74-76]. This uncertainty induced by the use of telemedicine could increase their use to confirm their diagnosis. There could also be an impact on follow-up visits and referrals to other health care providers and on modification of the relationship between the health care provider and the patient (therapeutic alliance) due to a first video or phone encounter. Telemedicine being an evolution in professional practices, quality standards and proper training of health care providers as well as the integration into the curriculum for trainees must also be anticipated to ensure the quality of remote care and promote the integration of telehealth [28,77,78]. Moreover, digital innovations, such as tools for clinical decision-making aids and improvements in information communication technologies, could compensate for the loss of hands-on assessment and could promote the acceptability of telemedicine by providing additional tools to help clinicians with evaluations and treatments [79-81].

Particular attention should as well be focused on the implementation of telemedicine that does not exclude populations, particularly in rural areas and low-income populations that may be affected by inequalities in access to information and communication technologies, such as access to reliable high-speed internet connections [82]. It is important to develop remote assessment methods using devices available to the public (smartphone and tablet) in contexts similar to clinical reality and not to neglect research of alternatives for populations without access to a high-speed internet connection, such as assessment by phone [60,66,67].

### Strengths and Limitations

Strengths of this review include the use of 4 major bibliographical databases, a comprehensive search strategy, the use of the validated QUADAS-2 score to assess methodological quality of included studies, and the use of the Grading of Recommendations, Assessment, Development and Evaluations approach to rate the certainty of the evidence. Prediction intervals were used to assess the impact of the heterogeneity on the results [45]. PRISMA (Preferred Reporting Items for Systematic Reviews and Meta-Analyses) guidelines were followed to ensure a robust review methodology [83]. Furthermore, this study is one of the first meta-analyses to pool concordance outcomes using novel validated statistical approaches [40,84].

However, some limitations should be highlighted. The presence of publication bias could not be assessed because there were <10 studies to conduct a funnel plot [85]. Our results highlighted that the accuracy of remote diagnoses was not influenced by the type of health care provider or the body region involved. However, the studies pooled in the meta-analyses included only orthopedic surgeons or physiotherapists to evaluate mostly adults with peripheral MSKDs. The accuracy of remote diagnoses made by other health care providers or in different clinical settings, such as rheumatology, or for spinal disorders, such as neck pain, could be different. Moreover, the included studies were carried out in experimental settings, which could overestimate the accuracy of the diagnoses compared to real-life conditions when participants are at home and not in a medical environment. Most of the studies (3/6, 50%) included in the meta-analyses used technologies developed specifically for telemedicine, ensuring an optimal quality of exchange between the evaluator and the participant. The limited number of studies included for treatment plans should also be highlighted. Therefore, more methodologically sound studies with larger sample size are needed.

### Conclusions

Health care providers can remotely make a diagnosis that is concordant with a usual in-person assessment for various MSKDs. Diagnostic concordance between remote and in-person assessments is good to very good, and future studies are unlikely to modify this conclusion. Type of health care provider, body region, technology used, and the presence of assistance with the patient do not seem to influence the accuracy of remote diagnoses, but more studies are needed to confirm the impact of such factors. Health care providers offer treatment plans that are consistent with those usually proposed in person. Treatment plan concordance between remote and in-person assessments is probably good to excellent, but future studies may modify these conclusions. There are still some considerations, such as the economic impact, modification of therapeutic alliance between health care provider and patient, or detection of serious pathologies and psychosocial risk factors, to investigate to fully appraise the challenges and benefits of the initial remote evaluation for patients with MSKDs. This systematic review with meta-analysis adds support for further development of hybrid or fully remote care pathways for patients with MSKDs to facilitate access to musculoskeletal care.

## Appendix

Multimedia Appendix 1 Search strategy.

Multimedia Appendix 2 Codes used in RStudio.

Multimedia Appendix 3 Manuscripts excluded with reasons.

Multimedia Appendix 4 Supplementary materials (characteristics of conference abstracts and subgroup analyses).

Multimedia Appendix 5 PRISMA 2020 checklist.

## Abbreviations

GRADE: Grading of Recommendations, Assessment, Development, and Evaluations
MSKD: musculoskeletal disorder
PABAK: prevalence-adjusted and bias-adjusted κ
PRISMA: Preferred Reporting Items for Systematic Reviews and Meta-Analyses
QUADAS-2: Quality Assessment of Diagnostic Accuracy Studies 2

## Data Availability

All analyses are available in Multimedia Appendix 4. The data sets generated during and analyzed during this study are available from the corresponding author on reasonable request.
