# Supplementary material for: Investigating the Use of Telemedicine by Health Care Providers to Diagnose and Manage Patients With Musculoskeletal Disorders: Systematic Review and Meta-Analysis
Source: J Med Internet Res. 2024 Sep 23;26:e52964. doi: 10.2196/52964 (PMC11459102; doi:10.2196/52964)
Supplement: Multimedia Appendix 1 [file jmir_v26i1e52964_app1.docx]

**Search Strategy**

Medline

| 1 | Telemedicine/ |
| --- | --- |
| 2 | telemedicine.ti,ab,kw. |
| 3 | mobile health.ti,ab,kw. |
| 4 | mhealth.ti,ab,kw. |
| 5 | telerehabilitation.ti,ab,kw. |
| 6 | tele-rehabilitation.ti,ab,kw. |
| 7 | ehealth.ti,ab,kw. |
| 8 | e-health.ti,ab,kw. |
| 9 | digital health.ti,ab,kw. |
| 10 | telehealth.ti,ab,kw. |
| 11 | tele-health.ti,ab,kw. |
| 12 | internet.ti,ab,kw. |
| 13 | telephone.ti,ab,kw. |
| 14 | skype.ti,ab,kw. |
| **15** | **or/1-14** |
| 16 | diagnosis/ |
| 17 | symptom assessment/ |
| 18 | diagnosis.ti,ab,kw. |
| 19 | diagnostic.ti,ab,kw. |
| 20 | range of motion.ti,ab,kw. |
| 21 | assessment.ti,ab,kw. |
| 22 | examination.ti,ab,kw. |
| 23 | checkers.ti,ab,kw. |
| 24 | accuracy.ti,ab,kw. |
| 25 | evaluation.ti,ab,kw. |
| 26 | device*.ti,ab,kw. |
| 27 | tool*.ti,ab,kw. |
| 28 | teleassessment*.ti,ab,kw. |
| 29 | outcome measurement.ti,ab,kw. |
| 30 | outcome measurements.ti,ab,kw. |
| 31 | consultation*.ti,ab,kw. |
| 32 | **or/16-31** |
| 33 | Musculoskeletal Pain/ |
| 34 | Back Pain/ |
| 35 | Neck Pain/ |
| 36 | Shoulder Pain/ |
| 37 | Shoulder Impingement Syndrome/ |
| 38 | Osteoarthritis, Knee/ |
| 39 | Osteoarthritis, Hip/ |
| 40 | Chronic Pain/ |
| 41 | (musculoskeletal* or musculo skeletal*).ti,ab,kw. |
| 42 | pain.ti,ab,kw. |
| 43 | ((neck or cervical* or shoulder* or elbow* or wrist* or thora* or lumba* or back or hip* or knee* or ankle*) adj2 (disorder* or syndrom* or osteoarthriti* or arthriti* or clinic*)).ti,ab,kw. |
| 44 | **or/33-43** |
| 45 | **15 AND 32 AND 44** |
| 46 | stroke.ti,kw. |
| 47 | neurology.ti,kw. |
| 48 | heart.ti,kw. |
| 49 | obesity.ti,kw. |
| 50 | vestibular.ti,kw. |
| 51 | Skin Diseases/ |
| 52 | patient satisfaction.ti. |
| 53 | children.ti. |
| 54 | mental health.ti. |
| 55 | review.ti. |
| 56 | survey.ti. |
| 57 | adolescents.ti. |
| 58 | cancer.ti,kw. |
| 59 | gynecologic.ti,kw. |
| 60 | copd.ti,kw. |
| 61 | urinary.ti,kw. |
| 62 | parkinson's disease.ti,kw. |
| 63 | bladder.ti,kw. |
| 64 | cardiovascular.ti,kw. |
| 65 | bowel.ti,kw. |
| 66 | endoscopy.ti,kw. |
| 67 | electrocardiogram*.ti,kw. |
| 68 | glucose.ti,kw. |
| 69 | dialysis.ti,kw. |
| 70 | ophthalmic.ti,kw. |
| 71 | diabetic.ti,kw. |
| 72 | smoker*.ti,kw. |
| 73 | pelvic pain.ti,kw. |
| 74 | chest pain.ti,kw. |
| 75 | palliative.ti,kw. |
| 76 | incontinence.ti,kw. |
| 77 | anesthesia/ |
| 78 | or/46-77 |
| 79 | 45 not 78 |
| 80 | 79 not (exp animals/ not humans.sh.) |
| 81 | limit 80 to yr="2000 -Current" |
|  | limit 81 to english language |

EMBASE

| 1 | Telemedicine/ |
| --- | --- |
| 2 | telemedicine.ti,ab,kw. |
| 3 | mobile health.ti,ab,kw. |
| 4 | mhealth.ti,ab,kw. |
| 5 | telerehabilitation.ti,ab,kw. |
| 6 | tele-rehabilitation.ti,ab,kw. |
| 7 | ehealth.ti,ab,kw. |
| 8 | e-health.ti,ab,kw. |
| 9 | digital health.ti,ab,kw. |
| 10 | telehealth.ti,ab,kw. |
| 11 | tele-health.ti,ab,kw. |
| 12 | internet.ti,ab,kw. |
| 13 | telephone.ti,ab,kw. |
| 14 | skype.ti,ab,kw. |
| **15** | **or/1-14** |
| 16 | diagnosis/ |
| 17 | symptom assessment/ |
| 18 | diagnosis.ti,ab,kw. |
| 19 | diagnostic.ti,ab,kw. |
| 20 | range of motion.ti,ab,kw. |
| 21 | assessment.ti,ab,kw. |
| 22 | examination.ti,ab,kw. |
| 23 | checkers.ti,ab,kw. |
| 24 | accuracy.ti,ab,kw. |
| 25 | evaluation.ti,ab,kw. |
| 26 | device*.ti,ab,kw. |
| 27 | tool*.ti,ab,kw. |
| 28 | teleassessment*.ti,ab,kw. |
| 29 | outcome measurement.ti,ab,kw. |
| 30 | outcome measurements.ti,ab,kw. |
| 31 | consultation*.ti,ab,kw. |
| 32 | **or/16-31** |
| 33 | Musculoskeletal Pain/ |
| 34 | Back Pain/ |
| 35 | Neck Pain/ |
| 36 | Shoulder Pain/ |
| 37 | Shoulder Impingement Syndrome/ |
| 38 | Osteoarthritis, Knee/ |
| 39 | Osteoarthritis, Hip/ |
| 40 | Chronic Pain/ |
| 41 | (musculoskeletal* or musculo skeletal*).ti,ab,kw. |
| 42 | pain.ti,ab,kw. |
| 43 | ((neck or cervical* or shoulder* or elbow* or wrist* or thora* or lumba* or back or hip* or knee* or ankle*) adj2 (disorder* or syndrom* or osteoarthriti* or arthriti* or clinic*)).ti,ab,kw. |
| 44 | **or/33-43** |
| 45 | **15 AND 32 AND 44** |
| 46 | stroke.ti,kw. |
| 47 | neurology.ti,kw. |
| 48 | heart.ti,kw. |
| 49 | obesity.ti,kw. |
| 50 | vestibular.ti,kw. |
| 51 | Skin Diseases/ |
| 52 | patient satisfaction.ti. |
| 53 | children.ti. |
| 54 | mental health.ti. |
| 55 | review.ti. |
| 56 | survey.ti. |
| 57 | adolescents.ti. |
| 58 | cancer.ti,kw. |
| 59 | gynecologic.ti,kw. |
| 60 | copd.ti,kw. |
| 61 | urinary.ti,kw. |
| 62 | parkinson's disease.ti,kw. |
| 63 | bladder.ti,kw. |
| 64 | cardiovascular.ti,kw. |
| 65 | bowel.ti,kw. |
| 66 | endoscopy.ti,kw. |
| 67 | electrocardiogram*.ti,kw. |
| 68 | glucose.ti,kw. |
| 69 | dialysis.ti,kw. |
| 70 | ophthalmic.ti,kw. |
| 71 | diabetic.ti,kw. |
| 72 | smoker*.ti,kw. |
| 73 | pelvic pain.ti,kw. |
| 74 | chest pain.ti,kw. |
| 75 | palliative.ti,kw. |
| 76 | incontinence.ti,kw. |
| 77 | anesthesia/ |
| 78 | or/46-77 |
| 79 | 45 not 78 |
| 80 | 79 not (exp animals/ not humans.sh.) |
| 81 | limit 80 to yr="2000 -Current" |
|  | limit 81 to english language |

Cochrane

| 1 | Telemedicine/ |
| --- | --- |
| 2 | telemedicine.ti,ab,kw. |
| 3 | mobile health.ti,ab,kw. |
| 4 | mhealth.ti,ab,kw. |
| 5 | telerehabilitation.ti,ab,kw. |
| 6 | tele-rehabilitation.ti,ab,kw. |
| 7 | ehealth.ti,ab,kw. |
| 8 | e-health.ti,ab,kw. |
| 9 | digital health.ti,ab,kw. |
| 10 | telehealth.ti,ab,kw. |
| 11 | tele-health.ti,ab,kw. |
| 12 | internet.ti,ab,kw. |
| 13 | telephone.ti,ab,kw. |
| 14 | skype.ti,ab,kw. |
| **15** | **or/1-14** |
| 16 | diagnosis/ |
| 17 | symptom assessment/ |
| 18 | diagnosis.ti,ab,kw. |
| 19 | diagnostic.ti,ab,kw. |
| 20 | range of motion.ti,ab,kw. |
| 21 | assessment.ti,ab,kw. |
| 22 | examination.ti,ab,kw. |
| 23 | checkers.ti,ab,kw. |
| 24 | accuracy.ti,ab,kw. |
| 25 | evaluation.ti,ab,kw. |
| 26 | device*.ti,ab,kw. |
| 27 | tool*.ti,ab,kw. |
| 28 | teleassessment*.ti,ab,kw. |
| 29 | outcome measurement.ti,ab,kw. |
| 30 | outcome measurements.ti,ab,kw. |
| 31 | consultation*.ti,ab,kw. |
| 32 | **or/16-31** |
| 33 | Musculoskeletal Pain/ |
| 34 | Back Pain/ |
| 35 | Neck Pain/ |
| 36 | Shoulder Pain/ |
| 37 | Shoulder Impingement Syndrome/ |
| 38 | Osteoarthritis, Knee/ |
| 39 | Osteoarthritis, Hip/ |
| 40 | Chronic Pain/ |
| 41 | (musculoskeletal* or musculo skeletal*).ti,ab,kw. |
| 42 | pain.ti,ab,kw. |
| 43 | ((neck or cervical* or shoulder* or elbow* or wrist* or thora* or lumba* or back or hip* or knee* or ankle*) adj2 (disorder* or syndrom* or osteoarthriti* or arthriti* or clinic*)).ti,ab,kw. |
| 44 | **or/33-43** |
| 45 | **15 AND 32 AND 44** |
| 46 | stroke.ti,kw. |
| 47 | neurology.ti,kw. |
| 48 | heart.ti,kw. |
| 49 | obesity.ti,kw. |
| 50 | vestibular.ti,kw. |
| 51 | Skin Diseases/ |
| 52 | patient satisfaction.ti. |
| 53 | children.ti. |
| 54 | mental health.ti. |
| 55 | review.ti. |
| 56 | survey.ti. |
| 57 | adolescents.ti. |
| 58 | cancer.ti,kw. |
| 59 | gynecologic.ti,kw. |
| 60 | copd.ti,kw. |
| 61 | urinary.ti,kw. |
| 62 | parkinson's disease.ti,kw. |
| 63 | bladder.ti,kw. |
| 64 | cardiovascular.ti,kw. |
| 65 | bowel.ti,kw. |
| 66 | endoscopy.ti,kw. |
| 67 | electrocardiogram*.ti,kw. |
| 68 | glucose.ti,kw. |
| 69 | dialysis.ti,kw. |
| 70 | ophthalmic.ti,kw. |
| 71 | diabetic.ti,kw. |
| 72 | smoker*.ti,kw. |
| 73 | pelvic pain.ti,kw. |
| 74 | chest pain.ti,kw. |
| 75 | palliative.ti,kw. |
| 76 | incontinence.ti,kw. |
| 77 | anesthesia/ |
| 78 | or/46-77 |
| 79 | 45 not 78 |
| 80 | limit 79 to english language |
| 81 | limit 80 to yr="2000 -Current" |

CINAHL

| S1 | MH (Telemedecine OR Telerehabilitation OR Telehealth OR "Cellular Phone" OR "Digital Technology" OR Internet OR "Internet-Based Intervention" OR Telephone OR Telecommunications OR Teleconferencing) |
| --- | --- |
| S2 | MH (Diagnosis OR Self-Diagnosis OR "Outcome Assessment" OR "Patient Assessment" OR "Clinical Assessment Tools" OR "Functional Assessment" OR "Range of Motion" OR "Physical Therapy Assessment" OR "Chiropractic Assessment" OR "Physical Examination" OR Validity OR Reliability OR Evaluation OR "Disability Evaluation" OR "Assistive Technology Devices" OR "Reference Tools" OR "Referral and Consultation" OR "Remote Consultation") |
| S3 | MH ("Back Pain" OR "Neck Pain" OR "Shoulder Pain" OR "Shoulder Impingement Syndrome" OR "Knee Pain" OR "Osteoarthritis, Knee" OR "Osteoarthritis, Hip" OR "Chronic Pain") OR TI("musculoskeletal*" OR "musculo skeletal*" OR "pain") OR TI((neck or cervical* OR shoulder* OR elbow* OR wrist* or thora* OR lumba* OR back OR hip* OR knee* OR ankle*) N2 (disorder* OR syndrom* OR osteoarthriti* OR arthriti* OR clinic*) AB("musculoskeletal*" OR "musculo skeletal*" OR "pain") OR AB((neck or cervical* OR shoulder* OR elbow* OR wrist* or thora* OR lumba* OR back OR hip* OR knee* OR ankle*) N2 (disorder* OR syndrom* OR osteoarthriti* OR arthriti* OR clinic*) |
|  | English only |
|  | Since 2000 |
|  | S1 AND S2 AND S3 |
