# Supplementary material for: Investigating the Use of Telemedicine by Health Care Providers to Diagnose and Manage Patients With Musculoskeletal Disorders: Systematic Review and Meta-Analysis
Source: J Med Internet Res. 2024 Sep 23;26:e52964. doi: 10.2196/52964 (PMC11459102; doi:10.2196/52964)
Supplement: Multimedia Appendix 2 [file jmir_v26i1e52964_app2.docx]

**Codes used in Rstudio**

#Install Matrix and metafor

install.packages(c("Matrix"))

library ("Matrix")

install.packages(c("metafor"))

library ("metafor")

#Import data manually

# Add variable names

names(#data_file_name) <- c("n","kappa*","95CIinf","95CIsup","var","w")

res <- rma(kappa*, var, data=data_file_name, slab=paste(ID))

res

#optional, assess influences etc

confint(res)

inf <- influence(res)

print(inf)

plot(inf)

#Forest plot

forest(res,

header="Authors, year n weight",

xlab="Kappa*",

slab=paste(ID),

xlim=c(-2,2), at=seq(0,1.2,by=0.1),

ilab=cbind(n, w), ilab.xpos=c(-0.89,-0.49)

)

*or PABAK
