# Supplementary material for: Investigating the Use of Telemedicine by Health Care Providers to Diagnose and Manage Patients With Musculoskeletal Disorders: Systematic Review and Meta-Analysis
Source: J Med Internet Res. 2024 Sep 23;26:e52964. doi: 10.2196/52964 (PMC11459102; doi:10.2196/52964)
Supplement: Multimedia Appendix 3 [file jmir_v26i1e52964_app3.docx]

Manuscripts excluded with reasons

No diagnostic or surgical triage agreement outcome (n=33)

1. Benger JR, Noble SM, Coast J, Kendall JM. The safety and effectiveness of minor injuries telemedicine. *Emerg Med J*. Jul 2004;21(4):438-45.

2. Bisson LJ, Komm JT, Bernas GA, et al. Accuracy of a computer-based diagnostic program for ambulatory patients with knee pain. *Am J Sports Med*. Oct 2014;42(10):2371-6. doi:10.1177/0363546514541654

3. Cabana F, Boissy P, Tousignant M, Moffet H, Corriveau H, Dumais R. Interrater agreement between telerehabilitation and face-to-face clinical outcome measurements for total knee arthroplasty. *Telemed J E Health*. Apr 2010;16(3):293-8. doi:10.1089/tmj.2009.0106

4. Chetty L. Telephone triage assessment for musculoskeletal disorders: part 2. *British Journal of Nursing*. 2012;21(22):1316-1320. doi:10.12968/bjon.2012.21.22.1316

5. Crawford AM, Lightsey HM, Xiong GX, et al. Interventional procedure plans generated by telemedicine visits in spine patients are rarely changed after in-person evaluation. *Reg Anesth Pain Med*. Jun 2021;46(6):478-481. doi:10.1136/rapm-2021-102630

6. Crawford AM, Lightsey HM, Xiong GX, Striano BM, Schoenfeld AJ, Simpson AK. Telemedicine visits generate accurate surgical plans across orthopedic subspecialties. *Arch Orthop Trauma Surg*. Nov 2022;142(11):3009-3016. doi:10.1007/s00402-021-03903-2

7. Cuesta-Vargas AI, Roldán-Jiménez C. Validity and reliability of arm abduction angle measured on smartphone: a cross-sectional study. *BMC Musculoskeletal Disorders*. 2016/02/20 2016;17(1):93. doi:10.1186/s12891-016-0957-3

8. Foster NE, Williams B, Grove S, Gamlin J, Salisbury C. The evidence for and against 'PhysioDirect' telephone assessment and advice services. *Physiotherapy*. Mar 2011;97(1):78-82. doi:10.1016/j.physio.2010.05.003

9. Ghorbani F, Kamyab M, Azadinia F. Smartphone Applications as a Suitable Alternative to CROM Device and Inclinometers in Assessing the Cervical Range of Motion in Patients With Nonspecific Neck Pain. *J Chiropr Med*. Mar 2020;19(1):38-48. doi:10.1016/j.jcm.2019.10.004

10. Goldstein Y, Schermann H, Dolkart O, et al. Video examination via the smartphone: A reliable tool for shoulder function assessment using the constant score. *J Orthop Sci*. Sep 2019;24(5):812-816. doi:10.1016/j.jos.2018.12.023

11. Gulle H, Prior T, Miller S, Birn-Jeffery AV, Morrissey D. Online questionnaire, clinical and biomechanical measurements for outcome prediction of plantar heel pain: feasibility for a cohort study. *Journal of Foot and Ankle Research*. 2021/04/26 2021;14(1):34. doi:10.1186/s13047-021-00472-w

12. Jansen T, Gathen M, Touet A, et al. Spine Examination during COVID-19 Pandemic via Video Consultation. *Zeitschrift fur Orthopadie und Unfallchirurgie*. 2021/04// 2021;159(2):193-201. doi:10.1055/a-1283-7160

13. Lightsey HMt, Crawford AM, Xiong GX, Schoenfeld AJ, Simpson AK. Surgical plans generated from telemedicine visits are rarely changed after in-person evaluation in spine patients. *Spine J*. Mar 2021;21(3):359-365. doi:10.1016/j.spinee.2020.11.009

14. Lopes F, Rodrigues M, Silva AG. User-Centered Development of a Mobile App for Biopsychosocial Pain Assessment in Adults: Usability, Reliability, and Validity Study. *JMIR Mhealth Uhealth*. May 14 2021;9(5):e25316. doi:10.2196/25316

15. Lyman S, Hidaka C, Fields K, Islam W, Mayman D. Monitoring Patient Recovery After THA or TKA Using Mobile Technology. *Hss j*. Dec 2020;16(Suppl 2):358-365. doi:10.1007/s11420-019-09746-3

16. Mallett R, Bakker E, Burton M. Is physiotherapy self-referral with telephone triage viable, cost-effective and beneficial to musculoskeletal outpatients in a primary care setting? *Musculoskeletal Care*. Dec 2014;12(4):251-60. doi:10.1002/msc.1075

17. Mani S, Sharma S, Singh DK. Concurrent validity and reliability of telerehabilitation-based physiotherapy assessment of cervical spine in adults with non-specific neck pain. *J Telemed Telecare*. Feb 2021;27(2):88-97. doi:10.1177/1357633x19861802

18. Montagnino J, Chen E, Wise A, McMullen C. Reliability of a Telemedicine Knee Exam. presented at: American Academy of Physical Medicine and Rehabilitation Annual Assembly 2021; 2021; https://pmrjabstracts.org/abstract/reliability-of-a-telemedicine-knee-exam/

19. Palacín-Marín F, Esteban-Moreno B, Olea N, Herrera-Viedma E, Arroyo-Morales M. Agreement between telerehabilitation and face-to-face clinical outcome assessments for low back pain in primary care. *Spine (Phila Pa 1976)*. May 15 2013;38(11):947-52. doi:10.1097/BRS.0b013e318281a36c

20. Pourahmadi MR, Bagheri R, Taghipour M, Takamjani IE, Sarrafzadeh J, Mohseni-Bandpei MA. A new iPhone application for measuring active craniocervical range of motion in patients with non-specific neck pain: a reliability and validity study. *Spine J*. Mar 2018;18(3):447-457. doi:10.1016/j.spinee.2017.08.229

21. Rothschild B. Telemedicine in Musculoskeletal Health: Adaptations, Efficacy, and Implications for Practice. *Hss j*. Oct 2021;17(3):360-361. doi:10.1177/15563316211040831

22. Sánchez-Rodríguez E, de la Vega R, Castarlenas E, Roset R, Miró J. AN APP for the Assessment of Pain Intensity: Validity Properties and Agreement of Pain Reports When Used with Young People. *Pain Med*. Oct 2015;16(10):1982-92. doi:10.1111/pme.12859

23. Scheidt S, Kehrer M, Jaenisch M, et al. A Feasibility Pilot Study on the Use of Telemedicine for the Examination of the Knee Joint. *Z Orthop Unfall*. Feb 2022;160(1):93-98. Eine Pilotuntersuchung zur Machbarkeit der Videosprechstunde bei muskuloskelettalen Beschwerden des Kniegelenks. doi:10.1055/a-1246-3615

24. Schoenfeld AJ, Davies JM, Marafino BJ, et al. Variation in Quality of Urgent Health Care Provided During Commercial Virtual Visits. *JAMA Intern Med*. May 1 2016;176(5):635-42. doi:10.1001/jamainternmed.2015.8248

25. Silva LB, Pereira DN, Chagas VS, et al. Orthopedic Asynchronous Teleconsultation for Primary Care Patients by a Large-Scale Telemedicine Service in Minas Gerais, Brazil. *Telemed J E Health*. Aug 2022;28(8):1172-1177. doi:10.1089/tmj.2021.0293

26. Slattery B, Ackerman L, Jagadamma KC. Service evaluation of telehealth in a physiotherapy musculoskeletal setting: Patient outcomes and results from risk stratification. *Musculoskeletal Care*. Dec 2022;20(4):977-990. doi:10.1002/msc.1623

27. Stenneberg MS, Busstra H, Eskes M, et al. Concurrent validity and interrater reliability of a new smartphone application to assess 3D active cervical range of motion in patients with neck pain. *Musculoskelet Sci Pract*. Apr 2018;34:59-65. doi:10.1016/j.msksp.2017.12.006

28. Truter P, Russell T, Fary R. The validity of physical therapy assessment of low back pain via telerehabilitation in a clinical setting. *Telemed J E Health*. Feb 2014;20(2):161-7. doi:10.1089/tmj.2013.0088

29. Uscher-Pines L, Mulcahy A, Cowling D, Hunter G, Burns R, Mehrotra A. Access and Quality of Care in Direct-to-Consumer Telemedicine. *Telemed J E Health*. Apr 2016;22(4):282-7. doi:10.1089/tmj.2015.0079

30. Van Der Veer SN, Beukenhorst AL, Ali SM, et al. Development of a Mobile Digital Manikin to Measure Pain Location and Intensity. *Stud Health Technol Inform*. Jun 16 2020;270:946-950. doi:10.3233/shti200301

31. Verzantvoort NCM, Teunis T, Verheij TJM, van der Velden AW. Self-triage for acute primary care via a smartphone application: Practical, safe and efficient? *PLoS One*. 2018;13(6):e0199284. doi:10.1371/journal.pone.0199284

32. Vuolio S, Winblad I, Ohinmaa A, Haukipuro K. Videoconferencing for orthopedic outpatients: one-year follow-up. *J Telemed Telecare*. 2003;9(1):8-11. doi:10.1258/135763303321159620

33. Waters L, Preston G, Nedungayil S. A retrospective review of the use of telephone and video consultations for a musculoskeletal service during COVID-19. *Physiotherapy*. 2022;114:e78. doi:10.1016/j.physio.2021.12.016

No comparison with in-person assesment (n=2)

1. Demmelmaier I, Denison E, Lindberg P, Asenlöf P. Physiotherapists' telephone consultations regarding back pain: a method to analyze screening of risk factors. *Physiother Theory Pract*. Oct 2010;26(7):468-75. doi:10.3109/09593980903433938

2. Sharma S, Shah R, Draviraj KP, Bhamra MS. Use of telephone interviews to follow up patients after total hip replacement. *J Telemed Telecare*. 2005;11(4):211-4. doi:10.1258/1357633054068883

Evaluator in the same room as the participant (n=1)

1. Owusu-Akyaw KA, Hutyra CA, Evanson RJ, Cook CE, Reiman M, Mather RC. Concurrent validity of a patient self-administered examination and a clinical examination for femoroacetabular impingement syndrome. *BMJ Open Sport Exerc Med*. 2019;5(1):e000574. doi:10.1136/bmjsem-2019-000574

Only healthy subjects included (n=1)

1. Mehta SP, Kendall KM, Reasor CM. Virtual assessments of knee and wrist joint range motion have comparable reliability with face-to-face assessments. *Musculoskeletal Care*. 2021;19(2):208-216. doi:https://doi.org/10.1002/msc.1525
