# Supplementary material for: Investigating the Use of Telemedicine by Health Care Providers to Diagnose and Manage Patients With Musculoskeletal Disorders: Systematic Review and Meta-Analysis
Source: J Med Internet Res. 2024 Sep 23;26:e52964. doi: 10.2196/52964 (PMC11459102; doi:10.2196/52964)
Supplement: Multimedia Appendix 4 [file jmir_v26i1e52964_app4.docx]

### ***SUPPLEMENTARY MATERIALS***

##### Table S1. Characteristics of conference abstracts on concordance studies (study settings).

| **Author & Year** | **Design** | **Country** | **Care setting** | **Modality of assessment** | **Technology** |
| --- | --- | --- | --- | --- | --- |
|  |  |  |  |  |  |
|  |  |  |  |  |  |
| Higginson et al., 2017 | Retrospective cohort | UK | Clinic | Synchronous | Telephone |
| Hutchinson et al., 2017 | Retrospective cohort | UK | Clinic | Synchronous | Telephone |

##### Table S2. Characteristics of conference abstracts on concordance studies (patient and evaluator characteristics..

| **Author & Year** | **Patients** | | | | **Evaluators** | | **Access to imaging tests to make Dx** |
| --- | --- | --- | --- | --- | --- | --- | --- |
|  | **MSKDs** | **n** | **Mean Age (SD)** | **Female (%)** | **Professions** | **Training** |  |
|  |  |  |  |  |  |  |  |
| Higginson et al., 2017 | Knee MSKDs | 135 | NR | NR | APPTs | NR | No |
| Hutchinson et al., 2017 | Spine MSKDs | 276 | NR | NR | APPTs | NR | No |

**MSKDs** Musculoskeletal disorders; **NR** Not reported; **APPTs** Advanced practice physiotherapists; **Dx** Diagnosis

Figure S1. Diagnostic concordance (Cohen’s Kappas and PABAKs) between in-person and remote assessment in patients with MSKDs in studies performed without assistance of a third party to evaluate the participant.

**
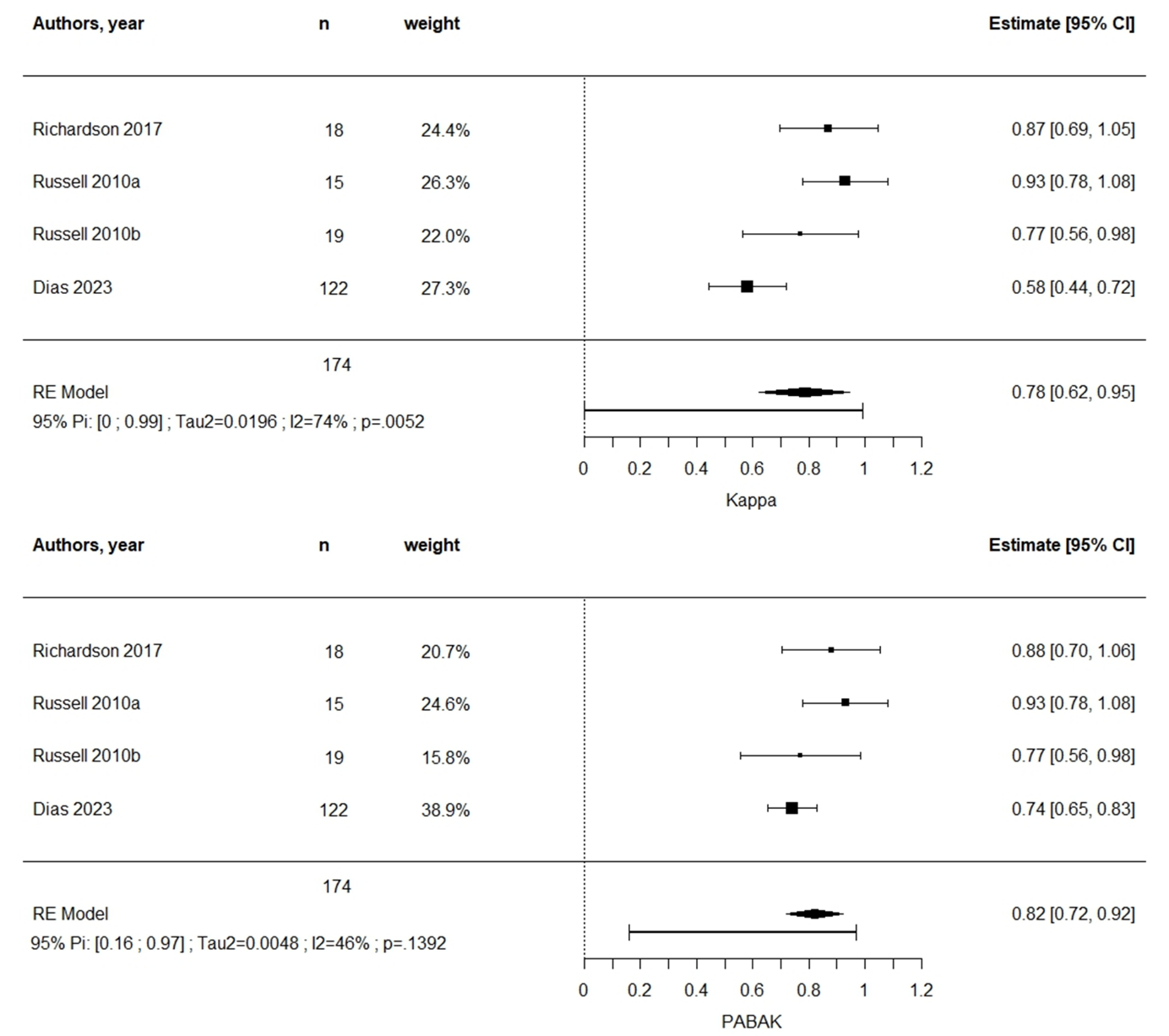
**

**95%CI:** 95% confidence interval; **95%Pi:** 95% prediction interval

**MSKDs:** Musculoskeletal disorders; **PABAK:** Prevalence-adjusted, bias-adjusted Kappa; **RE:** Random effect model; **Tau2/I2/p:** Test for heterogeneity

**Meta-Analysis with pooled Cohen’s Kappas** included studies with physiotherapists (n=6), physiotherapy students (n=3) and orthopedic surgeons (n=17) as evaluators

**Meta-Analysis with pooled PABAKs** included studies with physiotherapists (n=6), physiotherapy students (n=3) and orthopedic surgeons (n=17) as evaluators


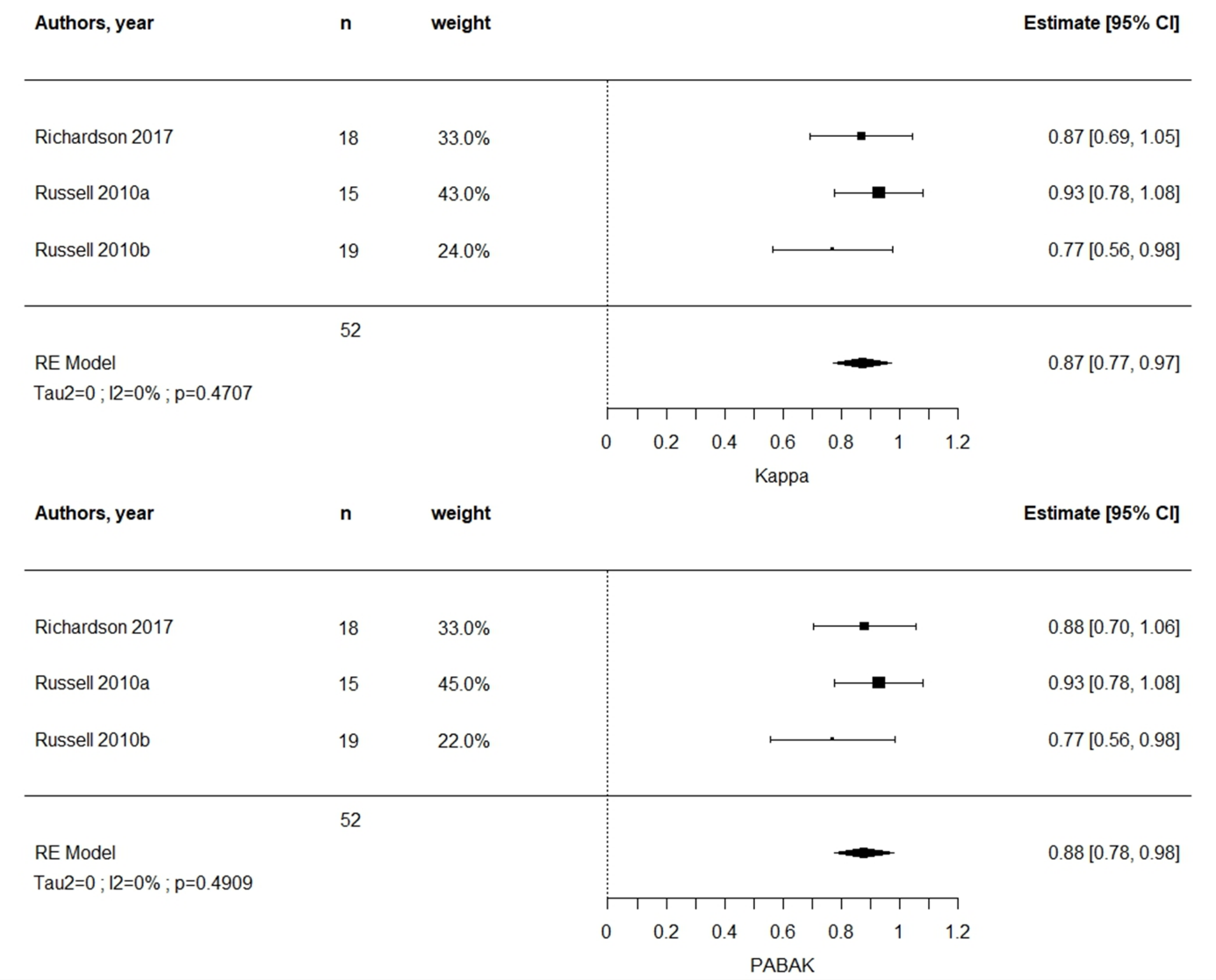
Figure S2. Diagnostic concordance (Cohen’s Kappas and PABAKs) between in-person and remote assessment in patients with lower limb MSKDs and only by physiotherapists.

**95%CI:** 95% confidence interval;

**MSKDs:** Musculoskeletal disorders; **PABAK:** Prevalence-adjusted, bias-adjusted Kappa; **RE:** Random effect model; **Tau2/I2/p:** Test for heterogeneity

**Meta-Analysis with pooled Cohen’s Kappas** included studies with physiotherapists (n=6) and physiotherapy students (n=3) as evaluators

**Meta-Analysis with pooled PABAKs** included studies with physiotherapists (n=6) and physiotherapy students (n=3) as evaluators


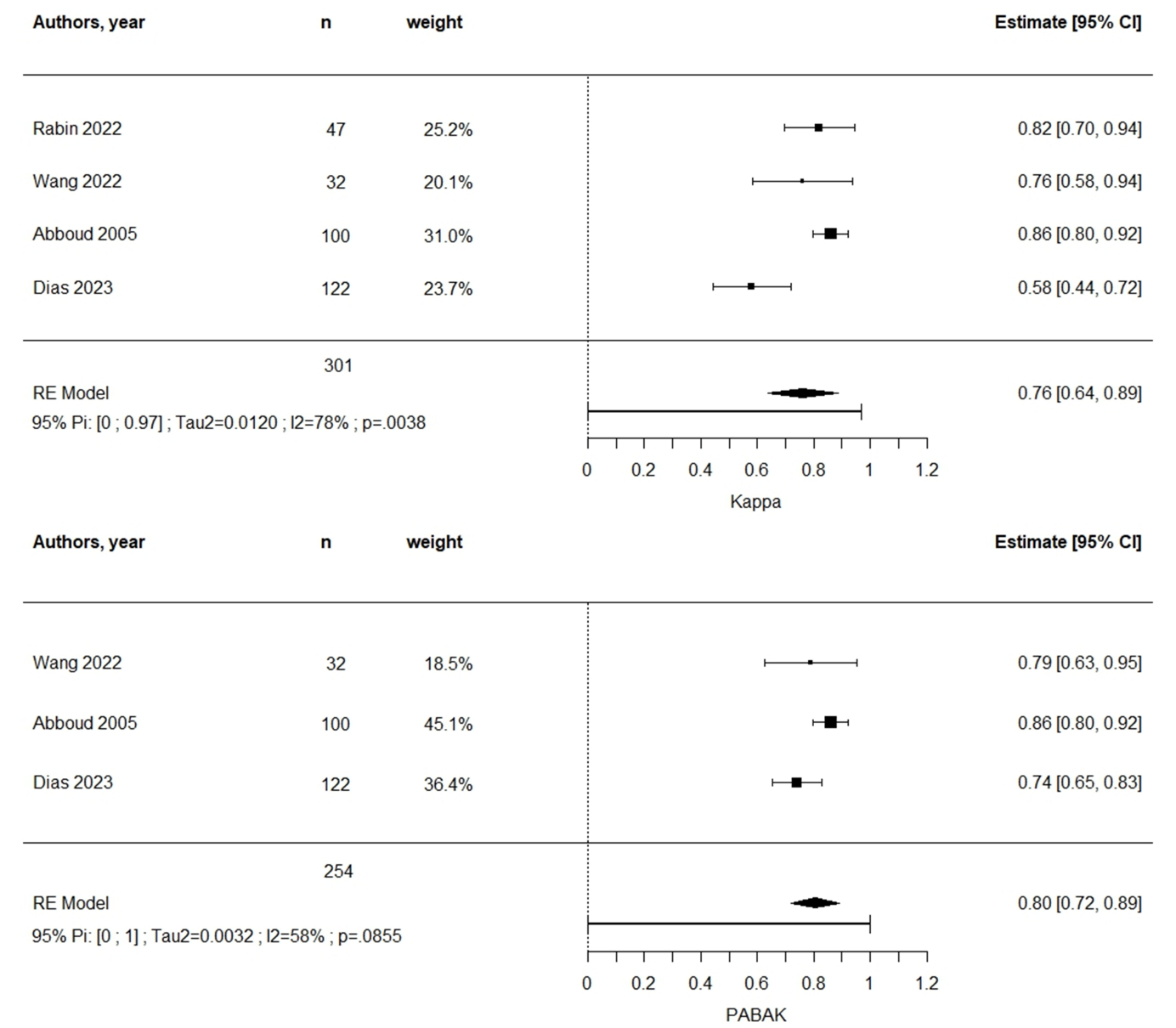
Figure S3. Diagnostic concordance (Cohen’s Kappas and PABAKs) between in-person and remote assessment only by orthopedic surgeons.

**95%CI:** 95% confidence interval; **95%Pi:** 95% prediction interval

**MSKDs:** Musculoskeletal disorders; **RE:** Random effect model; **Tau2/I2/p:** Test for heterogeneity

**Meta-Analysis with pooled Cohen’s Kappas** included studies with orthopedic surgeons (n=23) as evaluators

**Meta-Analysis with pooled PABAKs** included studies with orthopedic surgeons (n=21) as evaluators

Figure S4. Diagnostic concordance (Cohen’s Kappas and PABAKs) between in-person and remote assessment in patients with upper limb MSKDs

**
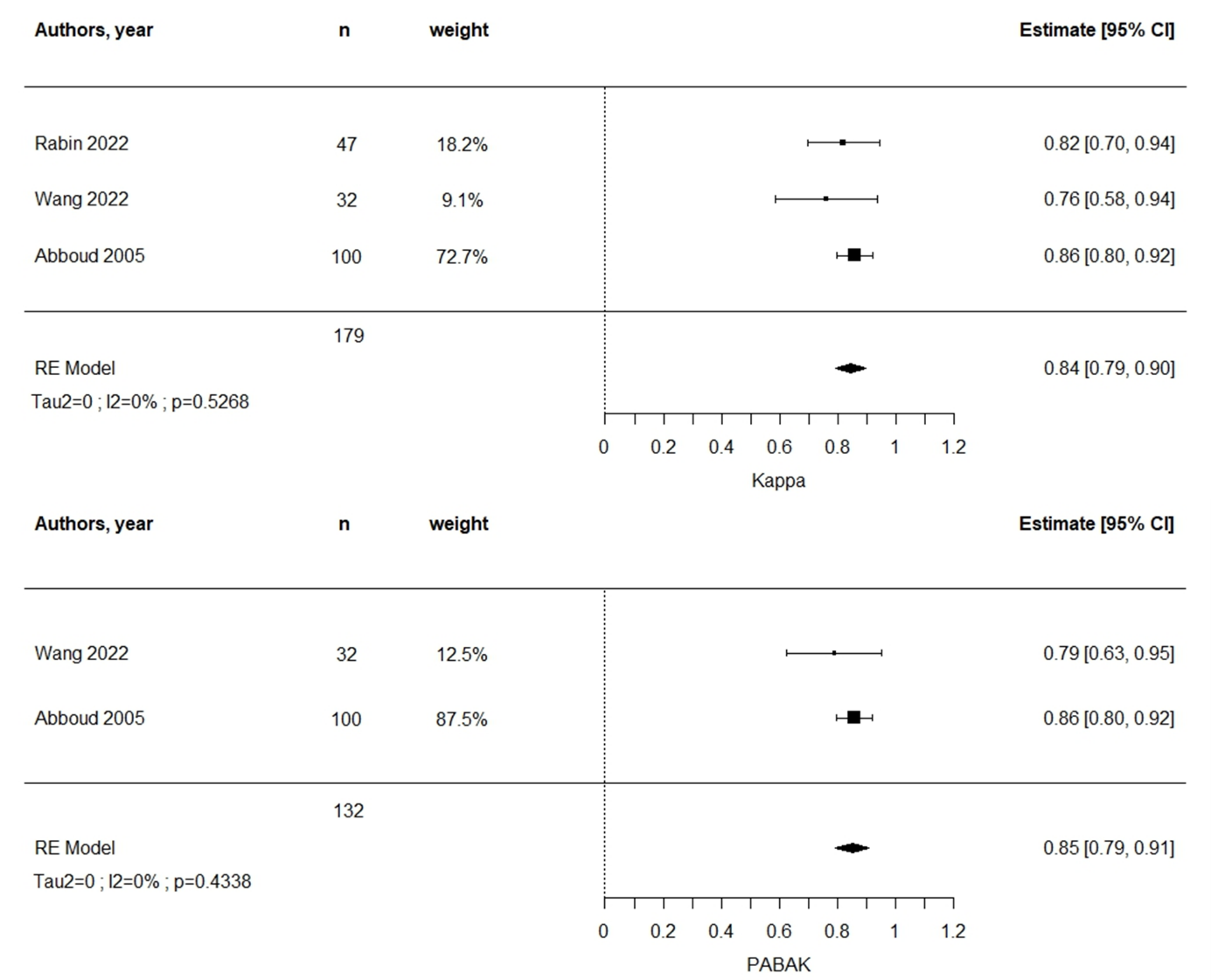
**

**95%CI:** 95% confidence interval;

**MSKDs:** Musculoskeletal disorders; **RE:** Random effect model; **Tau2/I2/p:** Test for heterogeneity

**Meta-Analysis with pooled Cohen’s Kappas** included studies with orthopedic surgeons (n=6) as evaluators

**Meta-Analysis with pooled PABAKs** included studies with orthopedic surgeons (n=4) as evaluators


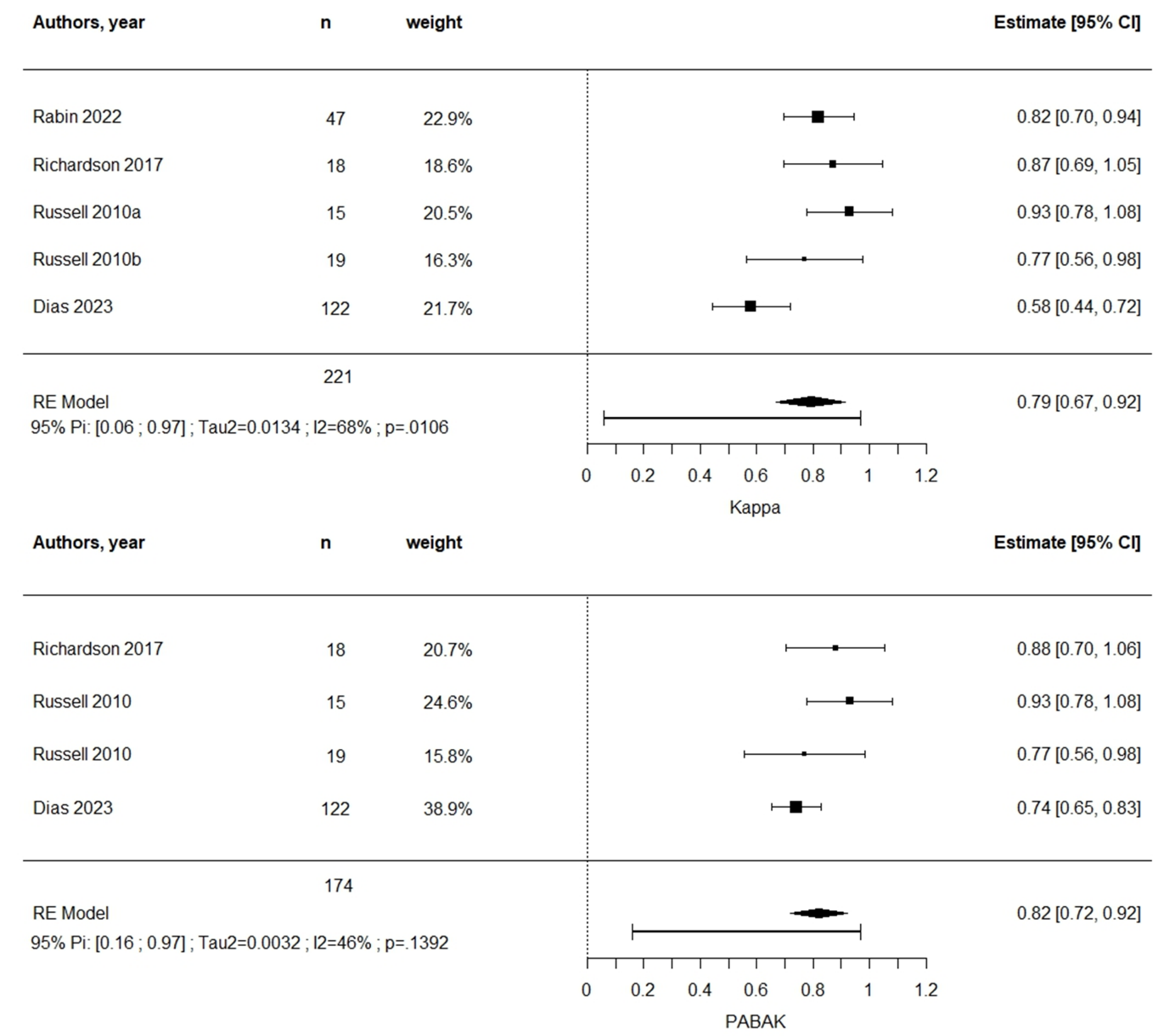
Figure S5. Diagnostic concordance (Cohen’s Kappas and PABAKs) between in-person and remote assessment in MSKDs patients, .studies using synchronous forms of telemedicine.

**95%CI:** 95% confidence interval; **95Pi:** 95% prediction interval

**MSKDs:** Musculoskeletal disorders; **PABAK:** Prevalence-adjusted, bias-adjusted Kappa; **RE:** Random effect model; **Tau2/I2/p:** Test for heterogeneity

**Meta-Analysis with pooled Cohen’s Kappas** included studies with physiotherapists (n=6), physiotherapy students (n=3) and orthopedic surgeons (n=19) as evaluators

**Meta-Analysis with pooled PABAKs** included studies with physiotherapists (n=6), physiotherapy students (n=3) and orthopedic surgeons (n=17) as evaluators

Figure S6. Diagnostic concordance (Cohen’s Kappas and PABAKs) between in-person and remote assessment in MSKDs patients, .studies using asynchronous forms of telemedicine.

**
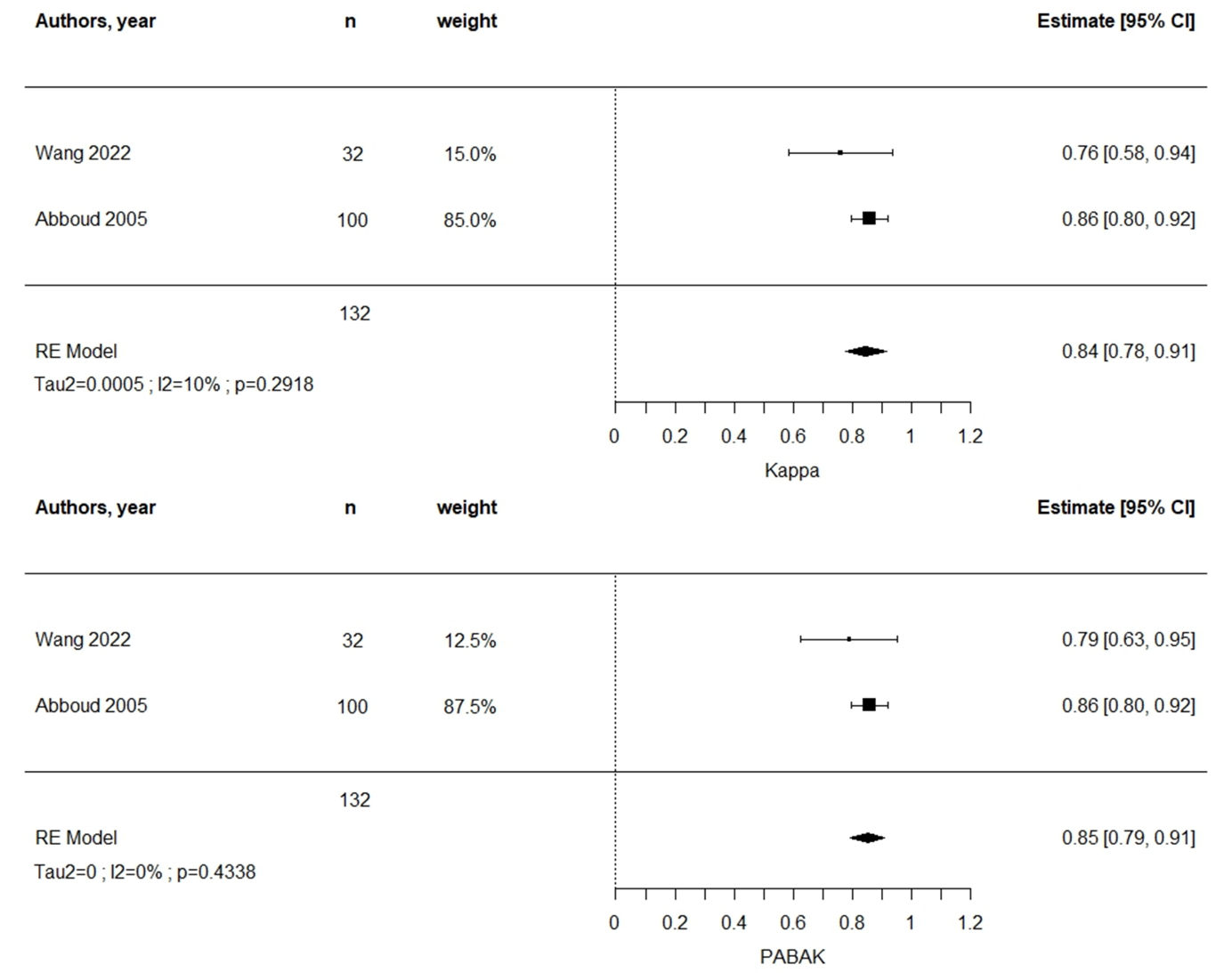
**

**95%CI:** 95% confidence interval;

**MSKDs:** Musculoskeletal disorders; **PABAK:** Prevalence-adjusted, bias-adjusted Kappa; **RE:** Random effect model; **Tau2/I2/p:** Test for heterogeneity

**Meta-Analysis with pooled Cohen’s Kappas** included studies with orthopedic surgeons (n=4) as evaluators

**Meta-Analysis with pooled PABAKs** included studies with orthopedic surgeons (n=4) as evaluators

Figure S7. Sensitivity analysis: diagnostic concordance (Cohen’s Kappas and PABAKs) between in-person and remote assessment in patients with MSKDs, studies with a QUADAS-2 without items evaluated at high risk of bias.

**
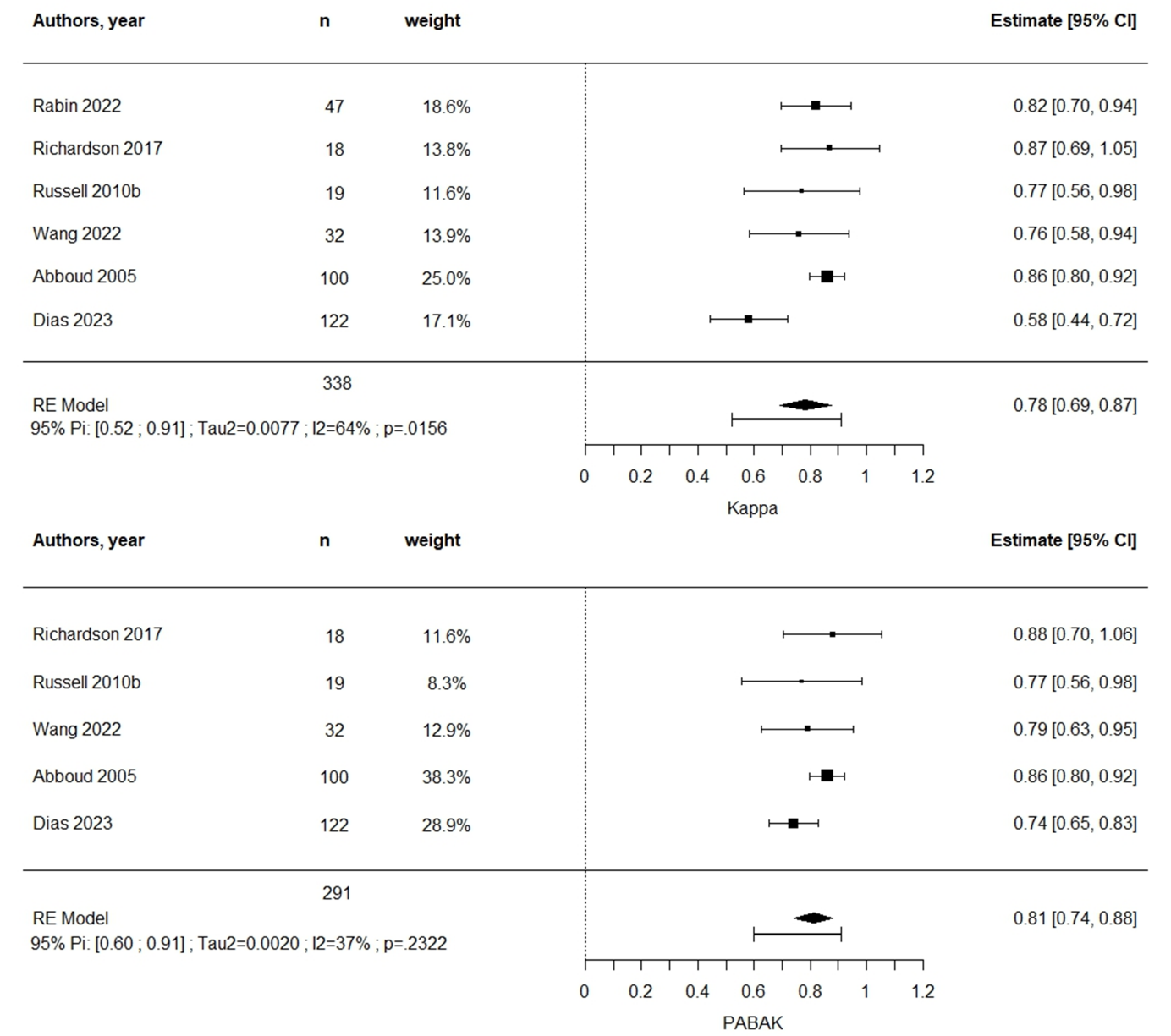
**

**95%CI:** 95% confidence interval; **95%Pi:** 95% prediction interval

**MSKDs:** Musculoskeletal disorders; **PABAK:** Prevalence-adjusted, bias-adjusted Kappa; **RE:** Random effect model; **Tau2/I2/p:** Test for heterogeneity

**Meta-Analysis with pooled Cohen’s Kappas** included studies with physiotherapists (n=6) and orthopedists (n=23)

**Meta-Analysis Cohen’s with pooled PABAKs** included studies with physiotherapists (n=6) and orthopedists (n=21)

**REFERENCES**

1. Higginson R and Hutchinson C. Diagnostic accuracy of knee pathologies by a telephone based Advanced Level Physiotherapy service. *Physiotherapy* 2017; 103: e104. DOI: 10.1016/j.physio.2017.11.080.

2. Hutchinson C and Higginson R. The diagnostic accuracy of spinal pathologies referred for by a telephone based advanced level physiotherapy triage service. *Physiotherapy* 2017; 103: e99. DOI: 10.1016/j.physio.2017.11.072.

3. Richardson BR, Truter P, Blumke R, et al. Physiotherapy assessment and diagnosis of musculoskeletal disorders of the knee via telerehabilitation. *Journal of Telemedicine and Telecare* 2017; 23: 88-95. DOI: 10.1177/1357633x15627237

4. Russell TG, Blumke R, Richardson B, et al. Telerehabilitation mediated physiotherapy assessment of ankle disorders. *Physiotherapy Research International* 2010; 15: 167-175. DOI: 10.1002/pri.471

5. Russell T, Truter P, Blumke R, et al. The diagnostic accuracy of telerehabilitation for nonarticular lower-limb musculoskeletal disorders. *Telemed J E Health* 2010; 16: 585-594. DOI: 10.1089/tmj.2009.0163

6. Dias JM, Jr., Mendes AF, Jr., Pestana de Aguiar E, et al. Interobserver Agreement and Satisfaction With the use of Telemedicine for Evaluating low Back Pain: A Primary, Observational, Cross-Sectional, Analytical Study. *Global Spine J* 2023: 21925682231194453. 20230816. DOI: 10.1177/21925682231194453

7. Rabin A, Dolkart O, Kazum E, et al. Shoulder assessment by smartphone: a valid alternative for times of social distancing. *Arch Orthop Trauma Surg* 2022; 142: 979-985. 20210113. DOI: 10.1007/s00402-021-03762-x

8. Wang G, Fiedler AK, Warth RJ, et al. Reliability and accuracy of telemedicine-based shoulder examinations. *J Shoulder Elbow Surg* 2022; 31: e369-e375. 20220510. DOI: 10.1016/j.jse.2022.04.005

9. Abboud JA, Bozentka DJ and Beredjiklian PK. Telemedicine consultation for patients with upper extremity disorders is reliable. *Clin Orthop Relat Res* 2005: 250-257. DOI: 10.1097/01.blo.0000155009.77961.5c
